# Transcriptional activity of the giant barrel sponge, *Xestospongia muta* Holobiont: molecular evidence for metabolic interchange

**DOI:** 10.3389/fmicb.2015.00364

**Published:** 2015-04-28

**Authors:** Cara L. Fiore, Micheline Labrie, Jessica K. Jarett, Michael P. Lesser

**Affiliations:** ^1^Department of Molecular, Cellular and Biomedical Sciences, University of New HampshireDurham, NH, USA; ^2^School of Marine Science and Ocean Engineering, University of New HampshireDurham, NH, USA

**Keywords:** ammonia oxidation, denitrification, B vitamins, sponge symbionts, metatranscriptome

## Abstract

Compared to our understanding of the taxonomic composition of the symbiotic microbes in marine sponges, the functional diversity of these symbionts is largely unknown. Furthermore, the application of genomic, transcriptomic, and proteomic techniques to functional questions on sponge host-symbiont interactions is in its infancy. In this study, we generated a transcriptome for the host and a metatranscriptome of its microbial symbionts for the giant barrel sponge, *Xestospongia muta*, from the Caribbean. In combination with a gene-specific approach, our goals were to (1) characterize genetic evidence for nitrogen cycling in *X. muta*, an important limiting nutrient on coral reefs (2) identify which prokaryotic symbiont lineages are metabolically active and, (3) characterize the metabolic potential of the prokaryotic community. *Xestospongia muta* expresses genes from multiple nitrogen transformation pathways that when combined with the abundance of this sponge, and previous data on dissolved inorganic nitrogen fluxes, shows that this sponge is an important contributor to nitrogen cycling biogeochemistry on coral reefs. Additionally, we observed significant differences in gene expression of the archaeal *amo*A gene, which is involved in ammonia oxidation, between coral reef locations consistent with differences in the fluxes of dissolved inorganic nitrogen previously reported. In regards to symbiont metabolic potential, the genes in the biosynthetic pathways of several amino acids were present in the prokaryotic metatranscriptome dataset but in the host-derived transcripts only the catabolic reactions for these amino acids were present. A similar pattern was observed for the B vitamins (riboflavin, biotin, thiamin, cobalamin). These results expand our understanding of biogeochemical cycling in sponges, and the metabolic interchange highlighted here advances the field of symbiont physiology by elucidating specific metabolic pathways where there is high potential for host-prokaryote interactions.

## Introduction

Marine sponges are widespread and ecologically important members of many marine benthic habitats (Diaz and Rutzler, 2001). Sponges also form symbiotic associations with a diverse community of prokaryotes (Simister et al., 2012). Sponges and their symbionts (the holobiont) perform a number of ecologically and biogeochemically important functions making them essential components of marine ecosystems (Taylor et al., 2007). For instance the ambient seawater that is pumped through sponges is modified as a result of physiological processes of both the host and symbiotic prokaryotes (Reiswig, 1971; Diaz and Ward, 1997; Lesser, 2006; Southwell et al., 2008; de Goeij et al., 2013); this phenomenon is particularly evident in high microbial abundance sponges (HMA; Hentschel et al., 2003; Weisz et al., 2008). Recent research on sponge microbial communities has characterized the community composition and diversity of sponge microbes from many marine habitats (Taylor et al., 2007; Simister et al., 2012; Fiore et al., 2013a; Kennedy et al., 2014). We now know “who is there” at the taxonomic levels of phyla and class for many shallow water demosponges (Webster et al., 2010; Schmitt et al., 2011). However, quantifying which of these symbiont lineages are metabolically active within the sponge is less well known (Kamke et al., 2010; Radax et al., 2012).

Our understanding of the functional roles that the prokaryotic symbionts facilitate within the sponge and how they interact with the host is also incomplete. Benefits for the host may derive from translocation of carbon rich organic compounds from symbionts, particularly photoautotrophic symbionts (Wilkinson, 1979; Arillo et al., 1993; Freeman and Thacker, 2011; Fiore et al., 2013b), although chemoautotrophic symbionts may supply small amounts of organic carbon as well (Hoffmann et al., 2009). Additionally, there is potential for transfer of nitrogen in either inorganic or organic forms from nitrogen-fixing symbionts to the host (Mohamed et al., 2008; Fiore et al., 2013b; Zhang et al., 2014). With evidence for specific benefits to the host sponge limited, Hoffmann et al. (2009) suggest that the main benefit to the sponge host to harbor and maintain a symbiotic microbial community is the removal of toxic compounds. Ammonia, nitrite, and nitrate could be detrimental to sponges at high concentrations and appear to be efficiently removed during processes of aerobic and anaerobic ammonia oxidation, nitrite oxidation, and denitrification (Hoffmann et al., 2009). These nitrogen transformations are also important in terms of their impact on the availability of dissolved inorganic nitrogen (DIN) and dissolved organic nitrogen (DON) to the surrounding environment (Diaz and Ward, 1997; Southwell et al., 2008; Hoffmann et al., 2009; Fiore et al., 2010, 2013b).

Quantifying the functional information (i.e., transcripts) of processes driven by sponge-associated prokaryotes and the environmental effects on those processes that influence both biogeochemical cycling and host-microbe interactions is essential to fully understand the multiple roles of sponges in benthic marine ecosystems. Recent studies aimed at understanding the taxonomic diversity and functional roles of sponge symbionts have utilized high throughput sequencing methods. These studies have led to the discovery of eukaryotic-like domains in symbiont-derived proteins (Thomas et al., 2010; Siegl et al., 2011) and novel metabolic diversity (Radax et al., 2012; Li et al., 2014; Moitinho-Silva et al., 2014), which have significantly advanced our understanding of the sponge-microbe relationship. Any understanding of the metabolic capabilities of sponge symbionts and the mechanisms of interaction with the sponge host is still preliminary, and in order to understand the ecological roles of sponges on coral reefs a characterization of symbiont metabolic potential and the interactions between symbionts and host is needed.

On Caribbean coral reefs, the giant barrel sponge *Xestospongia muta* is an abundant and ecologically important HMA sponge with a diverse symbiotic prokaryotic community (McMurray et al., 2008; Montalvo and Hill, 2011; Fiore et al., 2013b;Montalvo et al., 2014). Experimental studies have shown that *X. muta* and its symbiotic prokaryotic community influence nitrogen cycling on coral reefs, serving as both a source and sink for DIN (Southwell et al., 2008; Fiore et al., 2013a). To better understand the microbially-mediated processes underlying the dynamics of nitrogen cycling in this ecologically important sponge, and to gain insight into other potentially important metabolic processes expressed by the holobiont, the RNA pool of the holobiont was sequenced. Three compartments consisting of host mRNA, prokaryotic mRNA, and prokaryotic rRNA were bioinformatically separated from *X. muta* individuals collected from coral reefs at each of three locations in the Caribbean. This work expands our understanding of the metabolic diversity and activity present in sponge symbionts and provides target areas for further investigation into microbe-microbe interactions and host-microbe interactions.

## Methods

### Sample collection

As part of the study described in Fiore et al. (2013b) replicate sponges (*N* = 3) were sampled at 15 m depth at ~09:00 from each of three locations: Rock Bottom Reef, Little Cayman, Cayman Islands (LC) (19°42′7.36″ N, 80°3′24.94″ W), North Perry Reef, Lee Stocking Island (LSI) (23°47′0.03″ N, 76°6′5.14″ W), Bahamas, and Conch Reef, Key Largo, FL (FL) (24°57′0.03″ N, 80°27′11.16″ W). Sponge pieces were cut from the top rim of the sponge (“pie slice” including both pinacoderm and mesohyl) and placed in a plastic bag with seawater in a cooler until reaching the laboratory 15–20 min later. Each sponge sample was then placed in RNAlater (Ambion, Grand Island, NY) or DNA buffer (20% dimethylsulfoxide, 0.25 M ethylenediaminetetraacetic acid [EDTA], saturated NaCl, pH 7.5; Seutin et al., 1991) and kept frozen until reaching the University of New Hampshire where they were maintained at −70°C. From these sponge samples one replicate from each location was used for the metatranscriptome study. The number of samples used for each analysis are as follows: all nine sponge samples were used in the gene specific analyses (*N* = 3 from each location) while one sponge sample from each location was used to generate the metatranscriptome. From the metatranscriptome dataset, the three sponges samples were pooled for assembly but were analyzed individually for EMIRGE analysis as described below.

### RNA extraction and sequencing

We used an approach similar to a protocol that simultaneously sequenced the eukaryotic and prokaryotic communities of soils (Ulrich et al., 2008). Total RNA was extracted from each sponge sample using the RNeasy mini kit (Qiagen, Valencia, CA) and DNase treated using the DNA free kit (Ambion). The resulting RNA concentration and quality was determined using a NanoDrop 2000 and a bioanalyzer (Agilent, Santa Clara, CA). Extractions with a RIN of at least 7.5 were selected (samples had RNA concentrations of 90–230 ng μl^−1^) and samples were sent to the University of Illinois (Urbana-Champaign) for library construction and sequencing. There the eukaryotic rRNA was removed from 1 μg of total RNA with Ribominus Eukaryote kit (Invitrogen) and library construction was performed using a TruSeq RNA Sample Prep kit (Illumina, San Diego, CA) per the manufacturer's instructions. The libraries from the three sponge samples plus six coral libraries treated exactly the same (Jarett, 2012) and were amplified with 10 cycles of PCR, quantified by qPCR, and then pooled in equimolar concentration and sequenced using one lane on an Illumina HiSeq 2000. Sequencing was performed for non-overlapping paired-end reads ~100 nt in length and an average insert size of 240 nt (yielding ~380 M reads).

Raw reads were quality filtered using FASTX-Toolkit (http://hannonlab.cshl.edu/fastx_toolkit) and Prinseq (Schmieder and Edwards, 2011). In FASTX-Toolkit, reads were filtered based on an average quality score of 30, a minimum length of 50 nt, and the adapter sequences were trimmed using scripts by De Wit et al. (2012). Almost all reads were observed to contain an “N” as the first nucleotide, so the first nucleotide was trimmed from all reads in Prinseq. Following this, reads from paired files were checked to remove any unpaired reads in FASTX-Toolkit using the paired-end script from De Wit et al. (2012). A summary of reads at each step from quality trimming to assembly is provided in Table 1.

**Table 1:** **Summary of reads from each sponge sample**.

**Sample**	**Raw reads**	**Post quality control**	**Total (paired and unpaired) reads for assembly in CLC**	**Total (paired) reads for trinity assembly**	**Contigs in CLC assembly**	**Contigs in trinity assembly**
FL	33,220,038	33,220,038	13,723,211	7,698,642	35,219 (33%)	643 (67%)
LC	41,774,018	41,774,018	8,384,447	6,663,140		
LSI	51,696,682	51,696,682	11,101,071	8,835,358		

*Reads were mapped to Silva 111 database prior to assembly in CLC Workbench and Trinity to remove rRNA reads, but only paired reads were used for Trinity. The percentage value shown with the number of contiguous sequences (contigs) in each assembly is the percent of reads that mapped back to the assembly*.

### Assembly of 16S ribosomal RNA sequences using emirge

Quality trimmed and filtered Fastq files were run through the program EMIRGE (Miller et al., 2011), which uses the expectation maximum algorithm to probabilistically reconstruct full-length ribosomal sequences using high throughput sequencing reads. The parameters and databases used in EMIRGE analysis were optimized for working with our dataset, while working within the range of the available computational power. Quality of sequence reads produced from EMIRGE optimization runs were assessed manually by checking for excessive “N”s or homopolymer runs and by BLAST alignments. The final parameters used included: a hand-curated SILVA 108 non-redundant SSU ribosomal database as the reference database, max read length of 100, insert size of 240, standard deviation of 48, minimum read depth of 1, and 40 iterations.

The abundance estimate from EMIRGE that was used is the NormPrior, which is a length-normalized abundance estimate determined by the EMIRGE algorithm (Miller et al., 2011). Each sequence produced by EMIRGE is the result of merging sequences with greater than 97% similarity together at each iteration to form an operational taxonomic unit (OTU). EMIRGE was used separately for each of the three sponge samples rather than pooling reads from all three samples.

The ribosomal sequences from the EMIRGE analysis were classified using the Ribosomal Database Project (RDP) classifier v. 2.5 (Wang et al., 2007) with a confidence threshold of 80%. All sequences with matches to unclassified Bacteria or unclassified Archaea were then manually queried against NCBI to better identify the sequences and to remove any sequences that matched to 18S or chloroplast sequences. A custom script was used to merge the EMIRGE output files with the results of the RDP classification. The EMIRGE generated sequences were deposited in the community cyberinfrastructure for advanced microbial ecology research and analysis (CAMERA; Sun et al., 2010) database under accession number CAM_P_0001214 and are now available through the iMicrobe database under the same accession number (http://data.imicrobe.us/project/view/128).

### Assembly and analysis of putative mRNA reads

Quality filtered reads from all three sponges were mapped against the SILVA 111 SSU and LSU nr reference databases (Quast et al., 2012) in CLC Genomics Workbench v6.0 (CLC bio, Boston, MA, USA). Default settings of mismatch cost = 2, insertion cost = 3, deletion cost = 3, length fraction = 0.5, similarity fraction = 0.8, were used and map randomly was chosen for non-specific match handling. Unmapped reads were saved as putative mRNA reads. The putative mRNA reads were assembled *de novo* in CLC Workbench with graph parameters of word size = 20, bubble size = 50, and minimum contig length = 200. The option to map reads back to contigs was used with the above mapping settings. Putative mRNA reads were also assembled *de novo* with Trinity (Grabherr et al., 2011) using the following settings: minimum contig length = 250, and minimum kmer coverage = 2. FastAnnotator (Chen et al., 2012) was used to annotate each assembly.

Putative mRNA (poriferan and prokaryotic) contiguous sequences (contigs) from the CLC Workbench assembly were used in a BLASTx search against the NCBI RefSeq protein database (Sayers et al., 2010) using the CAMERA portal, and the top 20 matches with an *E*-value of 10^−4^ were saved and this setting was used in the rest of the BLAST searches. MEGAN (v4; Huson et al., 2007) was then used to visualize the BLASTx search results to obtain taxonomic information on the reads, as well as KEGG pathway classification information (Kanehisa and Goto, 2000; Kanehisa et al., 2011). The Least Common Ancestor (LCA) algorithm in MEGAN was used to classify contigs as poriferan or prokaryotic in origin (Min Support = 5, Min Score = 50, Top Percent = 10, Win Score = 0, Min Complexity = 0.44). In order to separate and compare the contigs of prokaryotic and poriferan origin, the identification number of contigs classified as Porifera (*n* = 7945) and as prokaryotic (*n* = 3723) were extracted in MEGAN and used to parse the original assembly file of contigs into separate files. These files were then individually used in BLASTx searches as described above and visualized in MEGAN. Contig sequences in all KEGG pathways that were examined were extracted and manually checked for quality by a BLASTx against NCBI database. A contig was discarded if it did not match to the expected gene based on the KEGG annotation. Phylogenetic information for each contig in a KEGG pathway was not available in MEGAN, therefore, prokaryotic contigs were classified by phyla if the top two matches were the same phyla and the query sequences was at least 50% homologous to the expected and top sequence. If this standard was not met, or if the top BLAST match was unclassified (e.g., marine bacterium), the contig was binned as unclassified. Poriferan contigs were confirmed if they matched to the sponge *Amphimedon queenslandica* using BLASTx, even if the homology was less than 50%. If poriferan contigs did not match to *A. queenslandica* they were discarded. In MEGAN, contigs were normalized to 100,000 per dataset for comparison between the putative sponge and prokaryotic mRNA datasets. Quality trimmed reads and associated metadata were deposited in the CAMERA database under accession number CAM_P_0001214 and are now available through the iMicrobe database under the same accession number (http://data.imicrobe.us/project/view/128).

### Characterization of genes involved in nitrogen cycling

Because of the role that sponges play in the cycling of DIN, the signature genes involved in nitrogen fixation (nitrogenase, *nif*H), nitrification (ammonia monooxygenase, *amo*A/*amoB*), denitrification (nitrite reductase, *nir*S; nitrate reductase, *nir*K; nitric oxide reductase, *qnor*B), and anaerobic ammonium oxidation (anammox, 16S rRNA genes) were selected as target amplification products. Genomic DNA was used as the template in these gene-specific reactions. However, for expression studies (i.e., QRT-PCR) of archaeal *amo*A genes, extracted RNA was used to synthesize cDNA.

Sponge samples collected as describe above (*n* = 3) and stored in DNA buffer were used in CTAB (hexadecyltrimethylammonium bromide) genomic DNA (gDNA) extractions along with water samples collected near the sponges at the same time. Seawater (~1 L) was collected adjacent to the same sponges described above and filtered through a 0.2 μm membrane filter (Whatman, PA, USA). Filters were cut in half (half archived) and also minced into smaller pieces to increase surface area for DNA extraction. Samples for both sponges and filters were processed as described in Fiore et al. (2013b).

Attempts to amplify genes using primers specific to bacterial *amo*A and *amo*B, *nir*S, and 16S rRNA genes of known anammox bacteria were not successful. Listed here are the references for the primers used and PCR protocols for those genes [*amo*A (Rotthauwe et al., 1997), *amo*B (Calvo et al., 2005; Junier et al., 2008), *nir*S (Braker et al., 1998), 16S rRNA Schmid et al., 2005; Shu and Jiao, 2008]. Successful amplification of *nif*H, *qnor*B, *nir*K, and archaeal *amo*A genes is described here. PCR for *nif*H genes was performed using two primer sets: OF1 (5′-ATXGTCGGXTGXGAXCCYAARGC-3′) and OR2 (5′-ATGGTGTTGGCGGCRTAZAKYGCCATCAT-3′) (X = C or T, Y = G or C, R = G or A, Z = C, G, or A, and K = G or T) (Olson et al., 1998), and a nested set modified from Zehr and McReynolds (1989) and Ohkuma and Kudo (1996): nifH3 (5′-ATRTTRTTNGCNGCRTA-3′) and IGK (5′-AARGGNGGNATHGGNAA-3′) followed by nifH1 (5′-TGYGAYCCNAARGCNGA-3′) and nifH2 (5′-ANDGCCATCATYTCNCC-3′) (IUPAC characters are used) similar to Olson and Lesser (2013). PCR for the nitric-oxide reductase gene, *qnor*B, was performed with the primers qnorB2F (GGNCAYCARGGNTAYGA) and qnorB7R (GGNGGRTTDATCADG) (Braker and Tiedje, 2003). Amplification of the nitrate reductase gene, *nir*K, was performed with the primers nirK1F (GGMATGGTKCCSTGGCA) and nirK5R (GCCTCGATCAGRTTRTGG) (Braker et al., 1998). For archaeal *amo*A genes, the primers Arch-amoAF (5′- STAATGGTCTGGCTTAGACG- 3′) and Arch-amoAR (5′-GCGGCCATCCATCTGTATGT- 3′) (Francis et al., 2005) were utilized based on methods described by Lopez-Legentil et al. (2010).

For all PCRs, three reactions of 25 μl were performed for each sample and pooled prior to electrophoresis. The PCR consisted of 0.25 μl of 50× Titanium Taq polymerase (Clontech, Mountain View, CA, USA), 2.5 μl of 10× Titanium Taq buffer, 0.2 mM dNTPs (Promega, Madison, WI, USA), 0.4 μM of each primer, and 25 ng of genomic DNA template. However, for the second step of the *nif*H nested protocol 1 μl of the first PCR step was used as template. Additional information on the amplification protocols for these genes is provided as supplemental information.

#### Cloning and phylogenetic analysis

The triplicate reactions were pooled and electrophoresed on a 1% agarose gel and bands of appropriate size were extracted using Qiaquick gel extraction kit (Qiagen, Valencia CA). Gel extracted products were cloned in *Escherichia coli* using the pGEM-T easy vector system (Promega, Madison, WI, USA) and JM109 chemically competent cells according to manufacturers instructions (Promega). Transformed cells were sent to Functional Biosciences Inc. (Madison, WI, USA) for sequencing on an ABI 3730xi DNA sequencer.

Chromatograms resulting from Sanger sequencing were manually checked for quality, and vector sequence was trimmed in the program Geneious v4.8.4 (Biomatters Ltd.). Trimmed sequences were then queried by BLAST against the NCBI nr database using Geneious and matching sequences were compiled. Pairwise alignments in Geneious were also performed for each sequence and its closest BLAST match, which were then translated and manually checked for errors. These sequences and the closest matches from BLAST queries were aligned (Geneious alignment algorithm with BLOSUM55 substitution matrix and default settings in Geneious) and the alignment was used to create a neighbor-joining tree in Geneious. *nif*H and *amo*A (see below) gene sequences were deposited in GenBank [accession numbers JQ912215 – JQ912238 (*nif*H), JQ912113 – JQ912214 (*amo*A), KJ020981 – KJ021003 (*qnor*B), KJ020980 (*nir*K)].

#### Characterization and quantitative expression of archaeal *amoA* genes

Total RNA was also extracted as described for the metatranscriptome analysis and converted to cDNA using RETROscript (Ambion) and random decamers following the manufacturer's protocol. The synthesized cDNA was used to quantify differential expression of archaeal *amo*A between the different locations. The archaeal *amo*A gene was chosen for quantitative expression analysis as this gene has previously been sequenced from *X. muta* (Lopez-Legentil et al., 2010) and because of its functional significance in the oxidation of ammonia. The presence and amplification of archaeal *amo*A gene numbers and transcripts (gDNA and cDNA as templates) was confirmed using PCR methods described above.

Quantitative real time PCR (QRT-PCR) was performed using genomic DNA for normalization of sponge samples from each location (Frias-Lopez et al., 2008). The QRT-PCR protocol and primers used were based on the study of Lopez-Legentil et al. (2010). Following a primer-efficiency experiment, 7.5 ng of gDNA or cDNA were added to 20 μl of SYBER green mix (Invitrogen, NY, USA) and a primer mix on a 96 well plate. The primer mix consisted of 1 μmol each primer and water to 200 μl; 135 μl of the primer mix was added to 225 μl of 2× SYBER green. Three replicates of gDNA and cDNA for each location were used along with replicate negative controls. The thermocycler protocol used was 50°C for 5 min, followed by 40 cycles of 95°C for 15 s, 50°C for 15 s, and 68°C for 0.5 s (Lopez-Legentil et al., 2010). Melt curve analysis was performed after the run to check for specificity of the PCR primers. Expression of cDNA samples were normalized to gDNA for each location separately and the **Δ**, ΔCt values were used to determine the difference in the relative expression between groups using the single sample with the highest **Δ**, ΔCt values (FL) as a calibrator in determining relative expression between the three locations. The calculated values were log + 1 transformed and tested for normality (Shapiro-Wilks; Royston, 1995) and homoscedasticity (O'Brien, 1981) prior to performing analysis of variance (ANOVA) and a Tukey's HSD *post-hoc* multiple comparison test. Statistical tests were performed using R (R Core Team) and JMP v 10.0.2.

## Results

### Taxonomic composition of the prokaryotic community based on assembled 16S rRNA sequences

**Figure 1:**
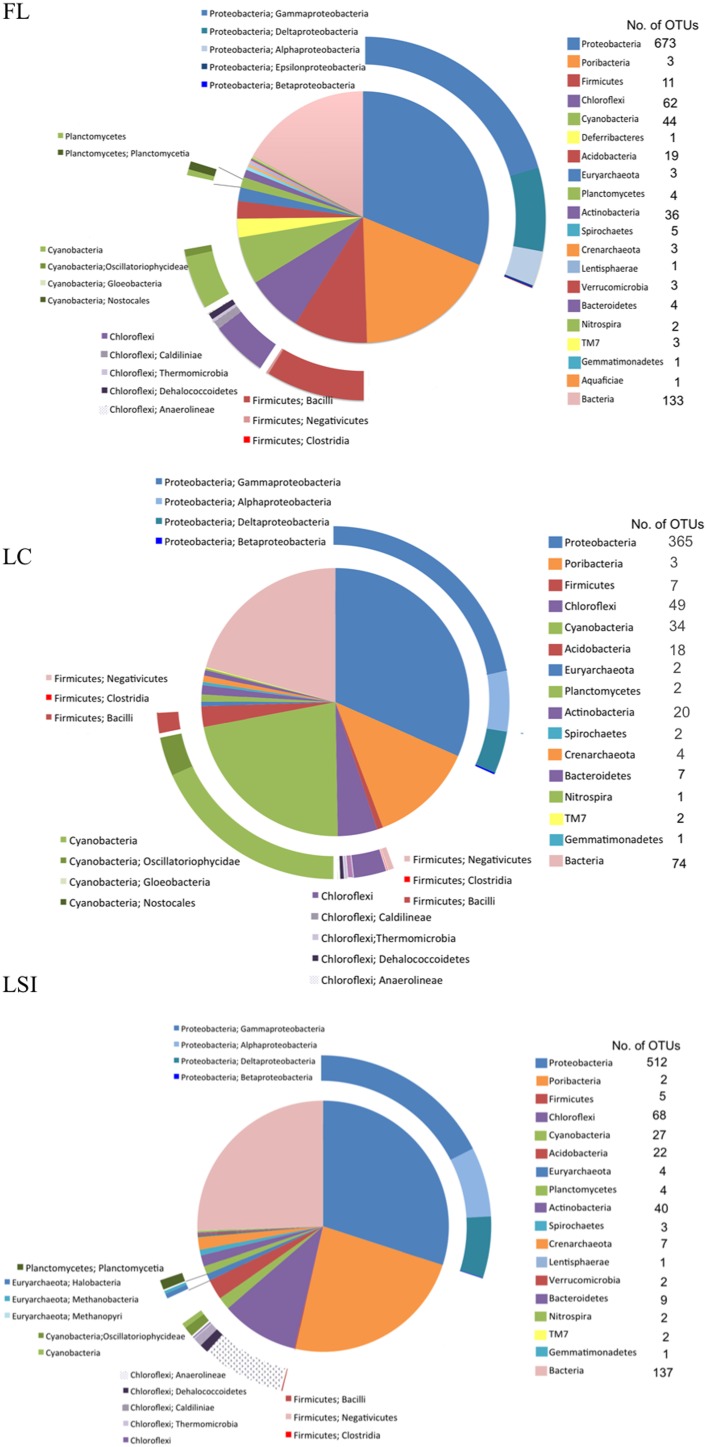
**Relative abundance of 16S ribosomal RNA operational taxonomic units (OTUs) generated by the program EMIRGE from total RNA short reads**. 16S rRNA OTUs were generated for one sponge from each location: Florida Keys (FL), Little Cayman (LC), and Lee Stocking Island, Bahamas (LSI). Phyla are listed in the clockwise order of the pie chart.

In all three sponge samples the most abundant OTUs (OTUs with the highest NormPrior), were assigned to the phylum Proteobacteria, and within this phylum, most were assigned to the class Gammaproteobacteria (Figure 1). The second most abundant group of OTUs for the FL and LSI sponges were assigned to the candidate phylum Poribacteria. In the LC sponge, Poribacteria classified OTUs were third most abundant behind Cyanobacterial OTUs (dominated by the genus *Synechococcus* spp). The green, non-sulfur, Chloroflexi, and Acidobacteria were also prominent OTUs in all three samples, although these varied in abundance among the three, with the highest abundance in the FL sponge compared to the LC and LSI sponges. The classes within these phyla also varied between the sponges, with Anaerolineae (Chloroflexi) more abundant in the LSI sponge than the FL and LC sponges. Other notable groups in terms of known functional activity include archaeal OTUs (Euryarchaeota and Thaumarchaeota), Nitrospirae and Planctomycetes, which were present in all sponge samples.

### Assembly of putative mRNA reads

The CLC Workbench assembly of non-ribosomal RNA reads yielded 35,219 contigs, or transcriptional features, with N50 = 1010, an average contig length of 979 bp, and GC content of 43% (Table 1). The Trinity assembly yielded 643 contigs, with N50 = 359, average contig length of 390, and GC content of 51%. Results of comparative analysis between the two assemblies are provided in the Supplemental Information (SI). As a result of that comparative analysis, the CLC generated *de novo* assembly was selected for use in the analyses described below. Of the CLC assembled contigs, 3,727 contigs were assigned to Bacteria or Archaea Domains, while 7,976 were assigned to the phylum Porifera based on the LCA algorithm results in MEGAN. Of the prokaryotic contigs, most were bacterial in origin (93%), while 7% were archaeal in origin.

### Functional profile of the prokaryotic community

Most of the assembled prokaryotic contigs (i.e., transcripts) were classified by KEGG as metabolism (~19%), followed by genetic information processing (~10%), and environmental information processing (~7%) (Figure S1). Cellular processing, organismal systems, and human disease represented less than 2% of transcripts, while most were unassigned (50%). Within metabolism, amino acid metabolism had the highest proportion of transcripts (~30%), followed by energy metabolism (~22%), carbohydrate metabolism (18%), nucleotide metabolism (17%), and metabolism of cofactors and vitamins (16%). The rest of the metabolism transcripts included lipid metabolism, xenobiotics metabolism, metabolism of other amino acids, glycan metabolism, biosynthesis of polyketides and terpenoids, and biosynthesis of other secondary metabolites (each <9%). More information on energy metabolism is provided in the Supplemental Information while transcripts involved in specific metabolic pathways of interest, such as nitrogen and sulfur cycling, and select biosynthetic pathways are described below.

#### Transcripts involved in nitrogen and other elemental cycling from the metatranscriptome

**Figure 2:**
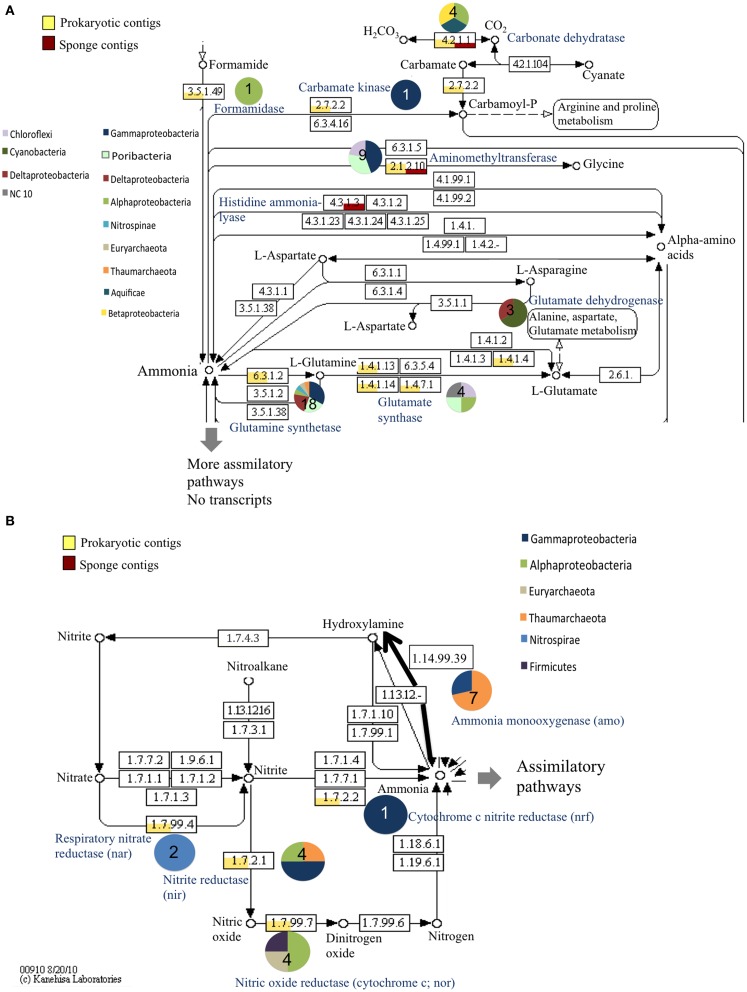
**Relative abundance of prokaryotic and sponge transcripts involved in nitrogen metabolism**. MEGAN was used to visualize transcripts on the KEGG map. The map was separated into assimilatory pathways **(A)** and mostly dissimilatory pathways **(B)** for visualization. Pie charts near each enzyme number indicate the phyla represented by the transcripts and the number in the pie chart is the number of transcripts. Enzyme names are provided in blue. For clarity not all intermediates are shown.

Many transcripts from the symbiotic prokaryotes matched to inorganic nitrogen cycling related genes by BLASTx including *nar* genes that encode a nitrate oxidoreductase (*Nitrospira fluvii*, 83% amino acid similarity), *nir*K genes encoding for nitrite reductases (Thaumarchaeota, 76%; *Oleispira antarctica*, 67%; *Mesorhizobium amorphae*, 72%), and an *nrf*A gene encoding for an ammonia-forming cytochrome c nitrite reductase (*Shewanella* spp., 58%, Figure 2). One transcript matched to a formamidase-encoding gene, which hydrolyzes formamide to formate and ammonia (*Nitratireductor aquibiodomus*, 78%). Other transcripts involved in nitrogen cycling were part of assimilatory pathways, with prokaryotic transcripts corresponding to reactions that incorporate ammonia into amino acids (e.g., glutamate synthase, glutamate dehydrogenase, glutamine synthetase, Figure 2), and sponge transcripts corresponding to reactions that metabolize amino acids (e.g., histidine ammonia-lyase, Figure 2). No transcripts involved in nitrogen fixation were recovered.

**Figure 3:**
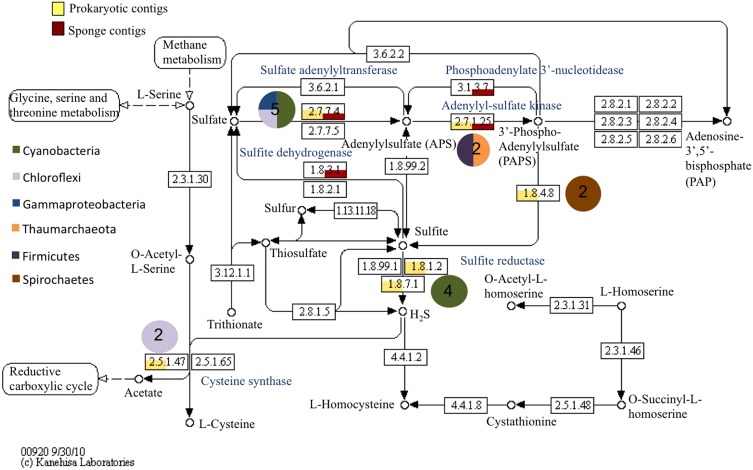
**Relative abundance of prokaryotic and sponge transcripts involved in sulfur metabolism**. MEGAN was used to visualize transcripts on the KEGG map. Pie charts near each enzyme number indicate the phyla represented by the transcripts and the number in the pie chart is the number of transcripts. Enzyme names are provided in blue.

Sulfur assimilation via different pathways in the host sponge and in the prokaryotic community was also observed (Figure 3). In the prokaryotic dataset, this included transcripts for the enzymes serine O-acetyltransferase (*Cellvibrio* sp., 58%; *Oceanicola granulosus*, 73%), cystathione beta-lyase (Chloroflexi, 58%), and sulfite reductase (*Synechococcus* sp., 65–70%). Sulfur assimilation related features from the host were mainly related to adenylylsulfate (APS) metabolism.

**Figure 4:**
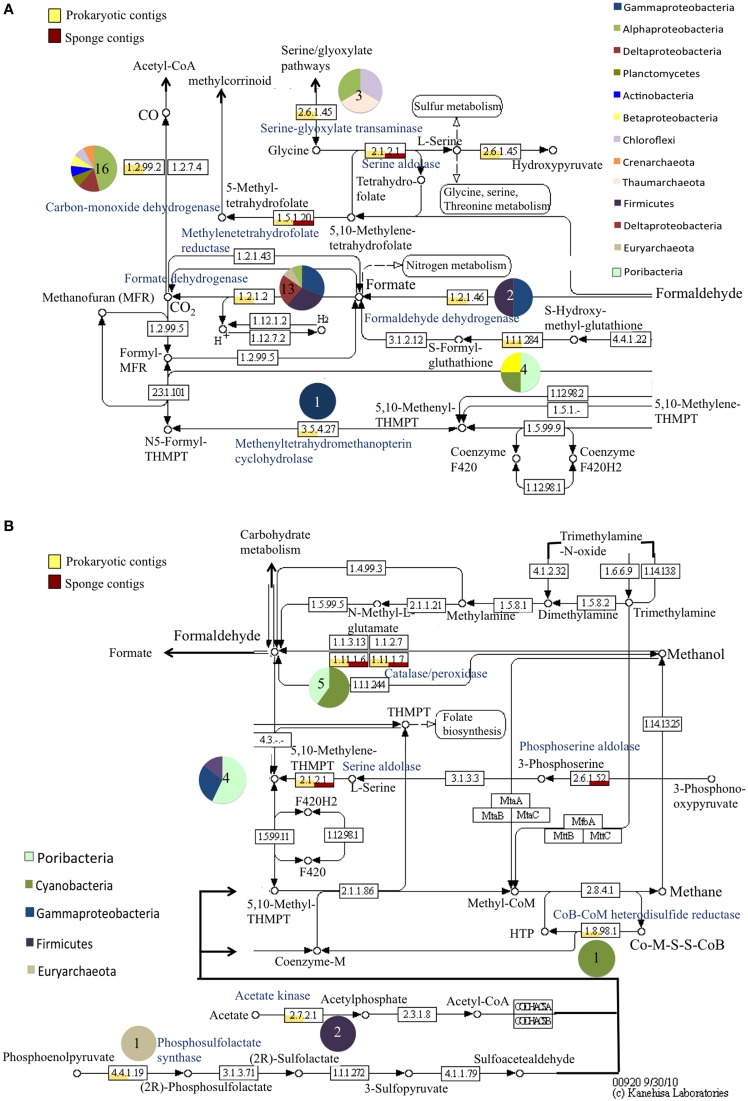
**Relative abundance of prokaryotic and sponge transcripts involved in methane metabolism**. MEGAN was used to visualize transcripts on the KEGG map. The map was separated into pathways centered on carbon monoxide and formate reactions **(A)** and methane reactions **(B)** for visualization. Pie charts near each enzyme number indicate the phyla represented by the transcripts and the number in the pie chart is the number of transcripts. Enzyme names are provided in blue. For clarity not all intermediates are shown.

Several transcripts were identified for single carbon (C_1_) compound metabolism, specifically formaldehyde, formate, carbon monoxide, and potentially methane (Figure 4). Methane related genes included the *hdr*B gene encoding for a heterodisulfide reductase (involved in coenzyme M regeneration; *Oscillatoria* sp., 48%; *Carboxydothermus hydrogenoformans*, 50%), a 5,10-methenyltetrahydromethanopterin cyclohydrogenase (*Methylophaga* sp., 56%), and a phosphosulfolactate synthase (*Methanothermus fervidus*, 42%; *Endozoicomonas montiporae*, 50%). Transcript matches to genes involved in formaldehye metabolism were recovered such as *frm*A, a glutathione-independent formaldehyde dehydrogenase, formate dehydrogenase (*fdh*), alcohol dehydrogenase enzymes, and many carbon monoxide dehydrogenase transcripts (*cdh*A) (Figure 4).

#### Characterization of nitrogen related genes by gene specific analysis

Gene sequences encoding the nitrogen fixing enzyme nitrogenase (*nif*H) (*n* = 10 from sponges, *n* = 14 from seawater) were successfully amplified from LC and LSI *Xestospongia muta* samples and from the water column. Sequences from FL were recovered, but were all low matches to the database and did not align with the other *nif*H sequences so were not analyzed further. The *nif*H-deduced amino acid sequences from *X. muta* that could be analyzed fell into cyanobacterial and proteobacterial groups (*nif*H Cluster I; Chien and Zinder, 1996) (Figure S2). The cyanobacterial *nif*H-deduced amino acid sequences from the current study were similar to, but distinct from those documented previously in sponges and corals (78–92%) (Mohamed et al., 2008; Olson et al., 2009). The cyanobacterial *nif*H-deduced amino acid sequences were also similar to *Xenococcus* sp. (87–89%), *Myxosarcina* sp. (87–89%) and the cyanobacterium UCYN-A (83–85%). Proteobacterial *nif*H-deduced amino acid sequences from the current study were most similar to either Alphaproteobacteria such as *Bradyrhizobium japonicum* (80–87%), or to Gammaproteobacteria such as *Vibrio* spp. (96–97%), and *Azotobacter chroococcum* (95–96%). Seawater-derived *nif*H-deduced amino acid sequences generally clustered together within each cluster, with the exception of sequence SW1.7 classified as Gammaproteobacteria, and sequence SW3.7, classified as Cyanobacteria.

Nitric oxide reductase gene (*qnor*B) sequences (*n* = 22) were successfully recovered from *Xestospongia muta* at all locations. Gene-deduced amino acid sequences (*qnor*B) recovered from *X. muta* were more similar to each other than to other known *qnor*B-deduced amino acid sequences from other bacteria and were ~36% similar to *Alcaligenes faecalis* (AM284323). One high quality sequence of the nitrate reductase gene, *nir*K, was recovered. Clone XmFL3m_3nirK (KJ020980) matched to several uncultured bacterial *nir*K genes as well as to *Pseudomonas aeruginosa* (AY345247, 63% amino acid similarity) and *Sinorhizobium* sp. (FJ598613, 63%).

**Figure 5:**
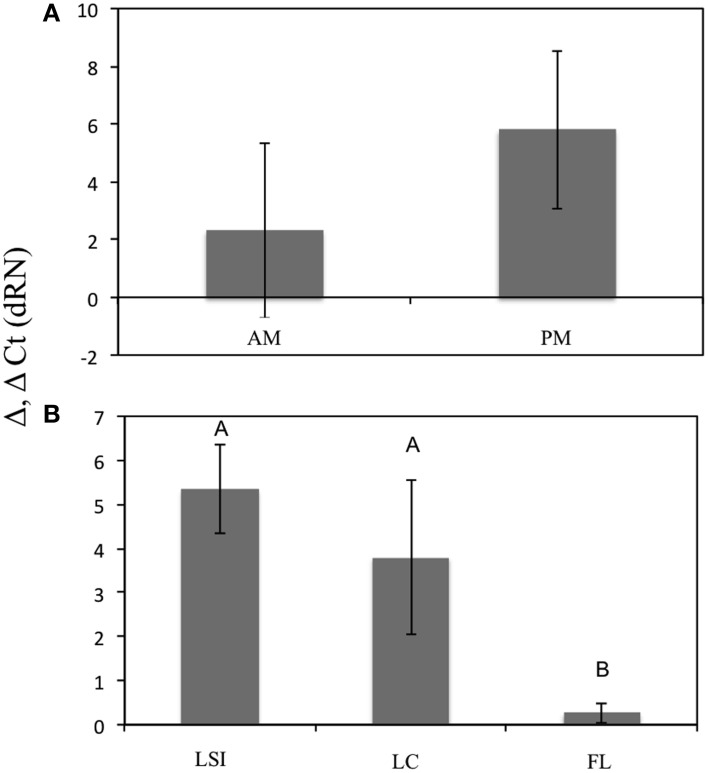
**Relative expression of archaeal *amo*A genes between geographic locations (A) and time points for LSI sponges only (B)**. Asterisk indicates significant difference [ANOVA, *F*_(2, 6)_ = 11.67, *p* = 0.0086; Tukey's HSD, *p* < 0.05].

The archaeal *amo*A-deduced amino acid sequences from *Xestospongia muta* were similar (all ~99%) to those documented previously by Lopez-Legentil et al. (2010) (data not shown). The QRT-PCR analysis showed a significant effect of location on the expression of *amo*A genes [*F*_(2, 6)_ = 11.67, *p* = 0.0086], with LSI and LC having significantly higher expression relative to FL (Tukey's HSD, *p* < 0.05) but no difference between each other (Tukey's HSD, *p* > 0.05) (Figure 5).

### Comparative analysis between host and symbionts

The most abundant gene ontology (GO) assigned terms were different between the metatranscriptome (prokaryotic transcripts) and the sponge transcriptome (Figure S3). While the most abundant GO biological process for both sets of transcripts was oxidation-reduction for the prokaryotic dataset electron transport, purine base metabolism, ribosome biogenesis and translation transcripts were the next most abundant. In the sponge dataset, signal transduction, protein phosphorylation, serine family metabolism and proteolysis were the next most abundant GO biological processes.

**Figure 6:**
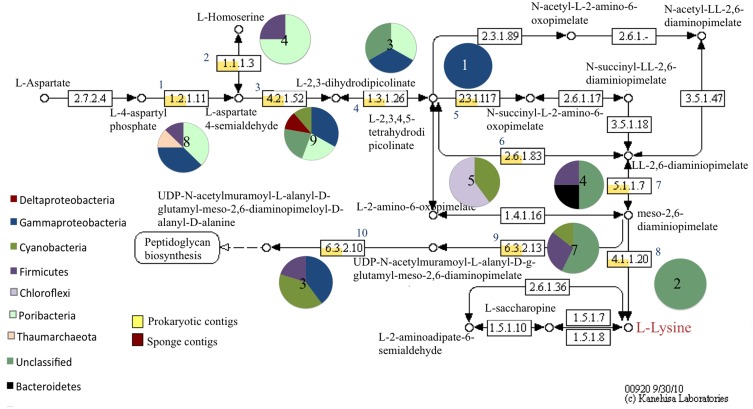
**Relative abundance of prokaryotic and sponge transcripts involved in lysine metabolism**. MEGAN was used to visualize transcripts on the KEGG map. Pie charts near each enzyme number indicate the phyla represented by the transcripts and the number in the pie chart is the number of transcripts. A subset of the map is shown for clarity. Numbers correspond to the following enzymes: (1) aspartate-semialdehyde dehydrogenase (2) homoserine dehydrogenase (3) L-aspartate-4-semialdehyde hydrolyase (4) 4-hydroxy-tetrahydrodipicolinate reductase (5) tetrahydropicolinate succinylase (6) LL-diaminopimelate aminotransferase (7) diaminopimelate epimerase (8) diaminopimelate decarboxylase (9) UDP-N-acetylmuramoyl-L-alanyl-D-glutamate-2,6,-diaminopimelate ligase (10) UDP-N-acetylmuramoyl-tripeptide-D-alanyl-D-alanine ligase.

The genome of *Amphimedon queenslandica* as annotated using the KEGG databases (Kanehisa et al., 2011) showed auxotrophy for the amino acids tryptophan, histidine, and lysine. Therefore, the biosynthetic pathways of these amino acids and of several vitamins were investigated further in the metatranscriptome dataset using MEGAN. Transcriptional features corresponding to genes in the biosynthetic pathway for chorismate, the precursor molecule to several aromatic compounds including amino acids, were prokaryotic in origin and included those encoding for the enzyme tryptophan synthase (e.g., *Endozoicomonas* sp., 58% homology; Figure S4). Additionally, prokaryotic and sponge-derived transcripts involved in tryptophan metabolism were characterized (Figure S5). Within the tryptophan metabolic pathway, transcripts were recovered that are involved in indole metabolism including, indole 3-pyruvate decarboxylase (unclassified bacterium, 57%), aldehyde dehydrogenase (e.g., *Endozoicomonas* sp., 87%), and nitrile hydratase (e.g., *Rubrobacter* sp., 73%) (Figure S5). The biosynthetic pathway of lysine was entirely represented by prokaryotic transcripts and included at least eight different phyla, notably, Poribacteria and Thaumarchaeota (Figure 6), while the biosynthetic pathway for histidine was represented by prokaryotic transcripts but also included several sponge transcripts (Figure S6). The sponge transcripts encoded histidine catabolism (Figure S6), including a histidine ammonia-lyase.

**Figure 7:**
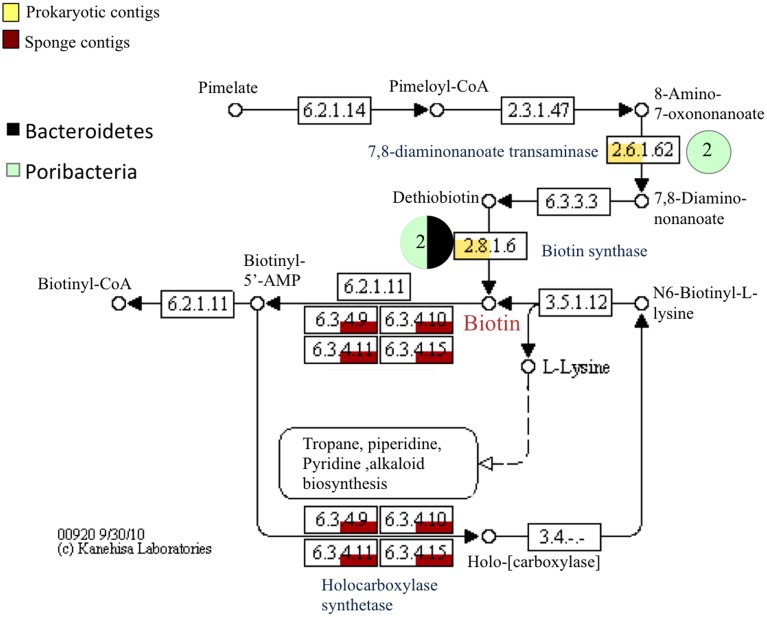
**Relative abundance of prokaryotic and sponge transcripts involved in biotin metabolism**. MEGAN was used to visualize transcripts on the KEGG map. Pie charts near each enzyme number indicate the phyla represented by the transcripts and the number in the pie chart is the number of transcripts. A subset of the map is shown for clarity. Enzyme names are shown in blue.

Transcripts involved in vitamin biosynthetic pathways included those for biotin, riboflavin, thiamin(e), and pantothenate. The biotin (vitamin B_7_) biosynthetic pathway was represented by poribacterial and bacteroidetes transcripts for 7,8-diaminonanoate transaminase (Poribacteria, 74 and 78%) and biotin synthase (e.g., Poribacteria, 90%; Figure 7). Sponge transcripts encoded the enzyme holocarboxylase synthetase. A similar trend was observed in the riboflavin (vitamin B_2_) biosynthetic pathway, but with more prokaryotic phyla involved (Figure S7). A key enzyme in riboflavin synthesis, riboflavin synthase, was represented by proteobacterial transcripts (e.g., *Endozoicomonas* sp., 71%). Both prokaryotic and sponge transcripts were involved in the conversions of FAD to/from riboflavin. Transcripts for the thiamin (vitamin B_1_) biosynthetic pathway included genes involved many early precursor compounds but also important genes such as *thi*G (Figure S8). No transcripts involved in the final steps leading to thiamin phosphate and thiamin were recovered, but sponge-derived transcripts did dominate the catabolism of thiamin. Prokaryotic transcripts corresponding to cobalamin (vitamin B_12_) biosynthesis were also recovered (Table S1).

In the host dataset, transporters for thiamin, riboflavin, amino acids, as well as organic solutes were recovered (Table S2). Host transcripts involved in the cellular responses (i.e., such as interleukin-1 receptor associated kinase 4-like (IRAK4) proteins, heat-shock proteins) of the host to stress and/or bacteria or viruses that were homologous to *A. queenslandica* predicted genes were recovered (see SI; Table S2). Eukaryotic-like domain containing proteins were detected in the metatranscriptome (prokayrotic) dataset, including two transcriptional features encoding leucine-rich repeat containing proteins, as well as tetratricopeptide repeat domains (TPR), and ankryin repeat domains (AR) (Table S1).

## Discussion

### Taxonomic characterization of the prokaryotic community

The giant barrel sponge, *Xestospongia muta*, is abundant on Caribbean coral reefs and has been shown to be an important mediator of inorganic nitrogen cycling on these coral reefs (e.g., Fiore et al., 2013b). There is also some evidence that the abundance of this sponge species is increasing (McMurray et al., 2010) and therefore understanding the metabolic potential of the prokaryotic community associated with *X. muta*, particularly as it relates to cycling of important nutrients such as nitrogen, should be fundamental to understanding the nutrient biogeochemistry of coral reefs. Here, we present the first analysis of the *X. muta* prokaryotic community using the simultaneous analysis of taxonomic (16S rRNA) and protein-coding (mRNA) genes within a metatranscriptome. As previously discussed there are limits to the interpretation of studies comparing rRNA and rDNA genes of prokaryotes to address simultaneously the question of taxonomy and metabolic activity (Kamke et al., 2010; Radax et al., 2012). The emergence of metatranscriptomes and their analysis on sponges (e.g., Moitinho-Silva et al., 2014) provides a much more comprehensive analysis of sponge symbiont metabolic activity by linking taxonomic identification with specific functions.

Overall there was large overlap in the community composition based on EMIRGE assembled ribosomal sequences and composition based on previous amplicon based pyrosequencing of 16S rDNA from *Xestospongia muta* (Fiore et al., 2013b). While only one individual was sampled from each location for this analysis, the relative abundance of prokaryotic phyla from the individual EMIRGE assembled datasets (one from each location) are more similar to the community derived from replicate sponge samples from each corresponding location (Fiore et al., 2013b) than the three EMIRGE datasets are to each other. As a result, while we provide preliminary support for potential differences in the “active” portion of the prokaryotic community in *X. muta* sponge between locations, the focus of the discussion is on implications derived from considering all three EMIRGE assembled datasets. The major phyla in both datasets included Proteobacteria, Cyanobacteria, Poribacteria, Chloroflexi, and Acidobacteria. Similarly, there were a higher proportion of cyanobacterial sequences recovered for the EMIRGE assembled rRNA in the LC samples relative to the FL and LSI samples, which is in agreement with the 16S rDNA analysis for *X. muta* (Fiore et al., 2013b). There were some notable differences, however, including a higher proportion of sequences recovered in this study representing Poribacteria, Firmicutes, and Planctomycetes, particularly in the FL and LSI samples, relative to the 16S rDNA analysis (Fiore et al., 2013b). This may be a result of primer mismatch (Radax et al., 2012) for members within these groups in the 16S rDNA study. While there are some discrepancies between the EMIRGE assembled 16S rRNA sequence dataset and the amplicon based 16S rDNA dataset, the results show a large overlap between the datasets similar to what has been observed in previous studies (Kamke et al., 2010; Radax et al., 2012).

The high proportion of sequences from Firmicutes, Gammaproteobacteria, Cyanobacteria, and Poribacteria suggests potential functional importance of these groups within the holobiont. Representatives from each of these groups, and the archaeal phylum Thaumarchaota, were identified as part of the core *Xestospongia muta* microbiome (Fiore et al., 2013b), providing support for the putative functional specialization of these groups within the holobiont. Cyanobacteria are well documented in *X. muta* (Steindler et al., 2005; Erwin and Thacker, 2007), with *Synechococcus* dominating the cyanobacterial community of this sponge (Erwin and Thacker, 2007). High relative abundance and high metabolic activity of this group based on transcripts indicates that sponge-derived cyanobacteria are major contributors to primary productivity on coral reefs where *X. muta* is abundant. Evidence for photoautotrophic symbiont activity in sponges and potential contribution to primary productivity is also provided by several other studies (e.g., Erwin and Thacker, 2008; Fiore et al., 2013a). Another well-documented group in sponges is the ammonia-oxidizing archaeal lineage of the Thaumarchoata (Hallam et al., 2006a; Lopez-Legentil et al., 2010). Thaumarchaeota constitute a substantial proportion of both the 16S rRNA sequences and transcripts in the metabolic pathway analysis, indicating that they are metabolically active within the sponge. Firmicutes, on the other hand, are a group that have been recovered from many sponges (e.g., Lee et al., 2009; Simister et al., 2012), but do not fall into a well-known functional category like Cyanobacteria and ammonia-oxidizing Archaea (AOA). Given the common occurrence of Firmicutes in both the EMIRGE analysis and transcript analysis in the current study, they represent a target group for further analysis in terms of co-evolution with its host and their functional diversity.

### Functional diversity of the prokaryotic community of *Xestospongia muta* and potential for host-microbe interactions

The results presented here represent the first description of the functional capabilities of the symbiotic prokaryotic community of the Caribbean sponge, *Xestospongia muta*. In nutrient limited environments such as shallow-water coral reefs, nutrients such as nitrogen are important in structuring microbial communities and supporting key reef members such as sponges and corals (Muscatine and Porter, 1977; D'Elia and Wiebe, 1990). Here, we discuss nitrogen cycling and metabolism of other relevant nutrients in the sponge based on (1) amplification of specific genes involved in DIN transformations from sponges for three locations in the Caribbean and, (2) characterization of specific pathways involving nutrient transformations from a metatranscriptome. We also utilize the *X. muta* metatranscriptome to discuss other functional aspects of symbiotic prokaryotic lifestyles using specific metabolic pathways of interest rather than focusing on the broader overview of the metatranscriptome, which been discussed in other sponge studies recently (Hoffmann et al., 2009; Li et al., 2014; Moitinho-Silva et al., 2014).

#### Evidence for complex nitrogen cycling in *Xestospongia muta*

Variability in fluxes of ammonium (NH^+^_4_) and nitrate + nitrite (NO^−^_x_) from *Xestospongia muta* led to the hypothesis that anaerobic nitrogen transformations occur in this sponge in addition to aerobic nitrification (Southwell et al., 2008; Fiore et al., 2013a). Additionally, nitrogen fixation, the process by which “new” nitrogen is generated from atmospheric nitrogen has been documented in other sponges (Wilkinson and Fay, 1979; Mohamed et al., 2008; Zhang et al., 2014) and we hypothesize that nitrogen fixation occurs in *X. muta*. Indeed, nitrogen fixation genes (*nif*H) were amplified from *X. muta* sponges. Proteobacerial and cyanobacterial *nif*H gene sequences were recovered, similar to what has been observed previously in sponges (Mohamed et al., 2008; Zhang et al., 2014). Seawater-derived *nif*H gene sequences were generally distinct from the sponge-derived sequences as observed in previous studies (Mohamed et al., 2008; Zhang et al., 2014). However, as some seawater and sponge-derived *nif*H sequences clustered together within major clusters; this may indicate that some of the prokaryotes within the sponge that were sampled were either transient or present as a result of feeding by the sponge. *nif*H transcripts were not observed in the metatranscriptome, likely indicating that at least at the time of sampling active nitrogen fixation was not occurring. Alternatively, with water being pumped almost constantly through the sponge and the release of NH^+^_4_ from the sponge as a waste product, there may not be strong pressure for the energetically expensive process of nitrogen fixation. That being said; high levels of DIN are not as strong as of repressor of nitrogen fixation as previously thought (Knapp, 2012).

Genes and transcripts from DNA-based amplification and from the metatranscriptome respectively did include several genes involved in other branches of the nitrogen cycle. Few clones were recovered from amplicon-based analysis of denitrification genes, particularly *nir*K genes. However, transcripts for respiratory nitrate reductase, nitrite reductase, and nitric oxide reductase enzymes were recovered from the metatranscriptome indicating that there is nitrate and nitrite reduction yielding denitrification intermediates such as nitrous oxide within *X. muta*. The low similarity between the amplified *qnor*B genes and other described *qnor*B genes and the discrepancy in recovery between the amplicon-based analysis and the metatranscriptome analysis likely indicates that more specific primers should be developed based on contig sequences to effectively amplify genes involved in denitrification in *Xestospongia muta*. In the metatranscriptome dataset, one transcript was recovered that encodes for a cytochrome c nitrite reductase, involved in dissimilatory nitrate reduction to ammonia (DNRA). The DNRA gene, *nrf*A, was also recovered in the metatranscriptome from a deep-sea sponge (Li et al., 2014), making this the first observation of this gene in a shallow water sponge. The presence of these transcripts help to explain the uptake of DIN by *X. muta* from a previous study (Fiore et al., 2013a), and indicates that these processes likely have an important role in mediating nitrogen cycling on coral reefs were *X. muta* is abundant.

Archaeal a*mo*A gene sequences and transcripts recovered from *Xestospongia muta* were similar to *amo*A sequences from other sponges, including previous studies on *X. muta* (Steger et al., 2008; Lopez-Legentil et al., 2010). Nitrification results in the production of NO^−^_3_ from NH_3_, and previous work found the volume-normalized flux of NO^−^_x_ was higher in the LSI sponges than the FL sponges (Fiore et al., 2013a), which could be a result of the higher activity of AOA in the LSI sponges. Accordingly, the expression of archaeal *amo*A genes was higher in the LSI and LC sponges than the FL sponges. What factors are driving the difference in expression of *amo*A as well as the differences in abundance of major taxa between sponges from the three locations is currently unknown. It is possible that higher dissolved organic carbon (DOC), corresponding to high DOM in the Florida Keys from allochthonous input (Boyer et al., 2006), could favor a mixotrophic lifestyle for AOA (Hallam et al., 2006b) and high DOC would also favor DNRA (Roberts et al., 2014).

Ammonia is also assimilated as a source of nitrogen for the prokaryotic community of *Xestospongia muta* (Fiore et al., 2013b). Analysis of transcripts related to nitrogen assimilation indicated that prokaryotic transcripts were involved in NH_3_ assimilation, while sponge transcripts present were involved catabolism of amino acids. This suggests that the main source of nitrogen for the sponge is heterotrophy; either dissolved organic nitrogen (DON) or particulate organic nitrogen (PON), or both, whereas the prokaryotic community obtains most of its nitrogen from NH_3_ via glutamine synthetase and glutamate synthase (GS-GOGAT) pathways. If there were transfer of nitrogen from symbionts to the sponge this would suggest that it may be in the form of amino acids or other nitrogenous compounds rather than as NH_3_. This notion is supported by the expression of genes encoding for the enzymes histidine ammonia lyase and aminomethyltransferase, which are involved in amino acid metabolism and NH_3_ formation, in the sponge dataset.

#### Carbon metabolism via methylotrophy by sponge-associated prokaryotes

The ability to utilize C_1_ compounds, such as methanol, formaldehyde, or carbon monoxide, as sources of carbon and energy has been characterized in diverse prokaryotic groups (Meyer and Schlegel, 1983; Oelgeschläger and Rother, 2008). Interestingly, genes encoding for a carbon monoxide dehydrogenase, which oxidizes carbon monoxide (CO) to carbon dioxide (CO_2_) were overrepresented relative to the bacterioplankton dataset in a metagenome of the sponge *Cymbastela concentrica* (Thomas et al., 2010), indicating that oxidation of CO may be an important source of energy for sponge symbionts. Additionally, transcripts representing diverse prokaryotic groups that corresponded to CO dehydrogenase were observed in the current study as well as several other metatranscriptome or metagenome studies (Fan et al., 2012; Radax et al., 2012; Li et al., 2014). In the current study, the transcripts encoding the oxidation and reduction of formaldehyde and formate were associated with the same pathway as CO dehydrogenase, indicating that methylotrophy is utilized by some sponge symbionts. Methylotrophy was reported previously in a sponge metatranscriptome (Moitinho-Silva et al., 2014), which suggests that this may be a common mechanism of carbon metabolism by sponge symbionts. In support of this, the recovery of a cyanobacterial heterodisulfide reductase gene in the present study suggests that methylotrophy by cyanobacteria, a common sponge symbiont group, may be occurring. Decreased concentrations of methane and production of hydrogen by cyanobacteria directly (Antal and Lindblad, 2005) or indirectly (Prasanna et al., 2002) has been documented in other systems (i.e., soil, cultures). Organisms exhibiting methylotrophy can be autotrophic or heterotrophic, facultative or obligate, aerobic or anaerobic, and most of the work characterizing the biochemistry of these organisms has been done with cultured bacteria (Chistoserdova et al., 2009). Coupling novel culturing approaches and focused genomic and transcriptomic analysis will be necessary to elucidate the physiology and ecology of methylotrophic organisms in sponges, and in particular, the potential methylotrophic activity by sponge-associated cyanobacteria.

#### Comparison of sponge and prokaryotic transcriptional features

Differences in metabolic function were apparent in comparing GO annotated transcriptional features in the category “biological process” in the sponge and prokaryotic datasets. Differences in the most abundant GO processes between the two datasets reflect the predominant metabolic functions of a metazoan eukaryote and a prokaryote, but may also highlight processes unique to the host or prokaryotic community. Interestingly, methylation and acyl-carrier-protein biosynthetic process are in the 15 most abundant processes for the prokaryotic metatranscriptome. Methylation of DNA can be a mechanism to resist incorporation of foreign DNA (Noyer-Weidner and Trautner, 1993) and may indicate more pressure for the symbiotic community to deal with foreign DNA than the host. Protein acylation is a common post-translational modification and can play a role in signaling (i.e., acyl-homoserine lactone molecules Parsek and Greenberg, 2000). This highlights the potential for future experimental studies examining the role of these two processes, methylation and acylation, in establishing and maintaining the symbiosis.

The range of processes observed in the sponge transcriptome (e.g., signal transduction, protein metabolism) are similar to what has been observed in the analysis of other sponge transcriptomes (Conaco et al., 2012; Riesgo et al., 2014) and while sponges as a basal taxon exhibit only tissue grade construction, many of the identified processes are highly conserved in metazoan physiology and molecular complexity (Nichols et al., 2006; Riesgo et al., 2014). The sponge dataset also yielded insight into the immunological activity of the sponge at the time of sampling. Many transcripts corresponding to a variety of innate immunity pathways were recovered and all of these have previously been observed in other sponge transcriptomes (Conaco et al., 2012; Riesgo et al., 2014). Some features could be a component of the response to invading bacterial cells, such as IRAK4 proteins (SI), which is involved in Toll-like receptor pathway signaling, while most are involved in several pathways that include responding to pathogens. Many transcripts were observed that are involved in apoptosis (e.g., caspases, AP-1), and this may be one mechanism by which the sponge processes senescent or infected cells. *Xestospongia muta* is probably responding to potentially foreign cells, yet whether these are “symbiont” cells, pathogens or opportunistic pathogens is not known. In corals, pattern recognition proteins involved in innate immunity are also involved in mediating initiation of symbiosis with *Symbiodinium* spp. (Kvennefors et al., 2010; Hamada et al., 2013), and there are likely to be similar interactions between sponges and their microbial symbionts (Hentschel et al., 2012).

Comparison of the prokaryotic and sponge transcriptional features in specific metabolic pathways has revealed novel information regarding the potential metabolic interactions between the sponge host and symbiotic prokaryotes. The production of amino acids, aromatic compounds, and vitamins specifically represent target pathways where synergistic interactions important to the establishment and maintenance of the symbiosis should be investigated. In *Xestospongia muta*, the amino acids, lysine, histidine, and tryptophan could be released by sponge symbionts and utilized by the sponge, as there are no characterized biosynthetic pathways in the *Amphimedon queenslandica* genome. For these amino acids prokaryotic transcripts were recovered that are directly involved in their biosynthesis, while both prokaryotic and sponge transcripts were present that are involved in the catabolism of these amino acids. Aromatic compounds in particular have been documented in other host-microbe interactions (Dillon and Dillon, 2004; Taprab et al., 2005) such as indole compounds are well documented in plant-microbe interactions (Spaepen et al., 2007) and algal-microbe interactions (Bagwell et al., 2014). Prokaryotic transcripts involved in the production of indole compounds via catabolism of tryptophan were recovered (e.g., indole-3-acetaldehyde, indole-3-acetamide), highlighting the potential for microbe-microbe interactions or microbe-host interactions via these molecules.

The notion that vitamins produced by sponge microbes could be beneficial to the host has been proposed previously (Thomas et al., 2010; Fan et al., 2012; Hentschel et al., 2012; Radax et al., 2012). The results described here also provide insight into specific vitamin biosynthetic pathways in the sponge holobiont that are relevant to understanding host-microbe intereactions. The biotin (vitamin B_7_) biosynthetic pathway was particularly striking, with transcripts corresponding to the key enzymes leading to biotin detected that were homologous to Poribacteria and Bacteroidetes genes. Genes involved in biosynthesis of B vitamins were documented in a poribacterial genome previously (Siegl et al., 2011). Biosynthesis genes for thiamin, vitamin B_1_, have also been detected in the archaeal symbiont, *Cenarchaeum symbiosum* (Hallam et al., 2006b). In the present study, transcripts corresponding to key thiamin biosynthesis genes were recovered including those corresponding *thi*G and *thi*C genes. However, the only sequences representing Thaumarchaeota corresponded to a cysteine desulfurase, which catalyzes an early step in thiamin biosynthesis. While no transcripts identifying genes leading to thiamin production were recovered, many of the key enzymes in the biosynthetic pathway were observed and sponge transcripts were recovered involved in the activation of thiamin (i.e., phosphorylation reactions). A similar pattern was observed for the riboflavin (vitamin B_2_) biosynthesis pathway, with prokaryotic transcripts corresponding to the production of riboflavin and sponge transcripts corresponding to the conversion between riboflavin and the cofactors flavin mononucleotide (FMN) and flavin adenine dinucleotide (FAD).

A recent study that examined the microbial communities of phylogenetically divergent sponges suggested that there might be selective pressure for vitamin producing prokaryotes (Fan et al., 2012). Vitamins including biotin, riboflavin, and thiamin, as well as amino acids derived from bacteria were shared with the host and between prokaryotes in the well-characterized tripartite symbiosis involving the insect glassy-winged sharpshooter and its symbionts a gamma-proteobacterium and a Bacteroidetes (McCutcheon and Moran, 2007). Additionally, members of Cyanobacteria, Alpha- and Gammaproteobacteria, and Bacteroidetes, as well as representatives of the Thaumarchaeota have been reported to be important producers of the vitamin cobalamin (B_12_) (Doxey et al., 2014; Sañudo-Wilhelmy et al., 2014). Transcripts for the genes encoding for cobalamin biosynthesis were recovered from the prokaryotic metatranscriptome in the current study and corresponded to lineages known to produce this vitamin. We propose that the pathways characterized here indicate that members of abundant lineages observed in the metatranscriptome interact with the sponge host via these vitamin and amino acid metabolites. This would suggest that the relationship is less commensal in nature, as has generally been considered for most sponge-associated microbes, and closer to mutualistic on the parasitic to mutualistic continuum (Lesser et al., 2013). Since many sponges, including *Xestospongia muta*, are heterotrophic filter feeders, they may be able to obtain these metabolites from their food but hosting prokaryotes that provide a continuous source of these important metabolites might be more advantageous and predictable in time and space in a relatively nutrient poor environment.

The approach described here has provided novel insights into the metabolic potential of *Xestospongia muta*-associated prokaryotes and potential points of interaction between the sponge and its symbiotic prokaryotes. Complementary methods of sequencing approaches along with physiological studies including metabolite studies are needed to effectively advance our understanding of host-microbe relationships in sponges. The metabolic pathways described here provide specific target areas for future experimental studies focused on sponge-associated microbes.

### Conflict of interest statement

The authors declare that the research was conducted in the absence of any commercial or financial relationships that could be construed as a potential conflict of interest.
