# Supplementary material for: Transcriptional activity of the giant barrel sponge, Xestospongia muta Holobiont: molecular evidence for metabolic interchange
Source: Front Microbiol. 2015 Apr 28;6:364. doi: 10.3389/fmicb.2015.00364 (PMC4412061; doi:10.3389/fmicb.2015.00364)
Supplement: Supplementary file 1 [file DataSheet1.DOC]

*Gene specific PCR protocols*

The thermocycler protocol for the nitric-oxide reductase gene, *qnor*B followed that by (Braker and Tiedje, 2003): 95°C 5 min followed by 40 total cycles: 10 cycles of 95°C 30 s, a touchdown from 57 to 52.5C 40s, and 72°C 1 min, and 30 cycles of the same series but with an annealing temperature of 55°C. Amplification of the nitrate reductase gene, *nir*K, included an initial denaturation of 5 min at 95°C followed by a “touchdown” PCR. This consisted of 30 total cycles of denaturation at 95°C 30 s, annealing for 40 s starting at 45°C and decreasing 0.5 C to 40°C (first 10 cycles) then remaining at 43°C, extension for 40 s at 72°C, followed by a final extension of 7 min at 72°C (Braker et al., 1998).

The thermocycler protocol for *nif*H primers OF1/OR2 primers was as follows: 95°C 5 min, 35 cycles of 95°C 30 s, 62°C 30 s, and 72°C 30 s, followed by 5 min at 72°C. The thermocycler protocol for nifhH3/IGK and nifH1/nifH2 primers was as follows: 95°C 5 min, 35 cycles of 95°C 1 min, 55°C 1 min, and 72°C 1 min, followed by 5 min at 72°C. The thermocycler protocol for *amo*A was as follows: 50°C for 30 min, 94°C for 2 min, 35 cycles of 94°C 15 sec, 53°C 30 sec, and 68°C 1 min, followed by 5 min at 72°C.

*CLC Workbench and Trinity assembly comparison:*

The CLC assembly yielded many more contiguous sequences (contigs) than the Trinity assembly; however, the number of reads that mapped back to the assembly, using Bowtie2 (Langmead and Salzburg, 2012), was substantially higher for the Trinity assembly (67%) than the CLC assembly (33%) (Table 2). This might be a result of chimeric sequences in the CLC assembly. Annotation of the CLC assembly yielded 50% of contigs matching a GO annotation and 68% of contigs matched to NCBI nr by BLASTx. Annotation of the Trinity assembly yielded 19% of contigs annotated with GO and 80% annotated by NCBI nr BLASTX matches. The CLC assembly was used as the contig number was higher and the annotation was approximately what we would expect in terms of percentage of contigs annotated and the classification of contigs by GO annotations. Due to potential chimeric sequences in the CLC assembly, all sequences in specific metabolic pathways were manually checked by BLASTx against the NCBI database for quality confirmation.

*Energy metabolism*

The energy metabolism of the symbiotic prokaryotes was of specific interest in this study with multiple transcripts involved in general energy metabolism (i.e., oxidative phosphorylation), photo- and chemoautotrophic metabolism, as well as nitrogen, sulfur, and methane cycling. Transcripts corresponding to NADH dehydrogenase (i.e., *nuo* genes), cytochrome oxidases (cytochrome c oxidase and cytochrome bd complex) and prokaryotic F-type ATPases were recovered from the prokaryotic dataset and are involved in oxidative phosphorylation. Transcripts assigned to the light reactions of photosynthesis included F-type ATPase and phostosystem proteins such as cp43 and cp47 (data not shown), and most of these (70%) had high homology to “*Candidatus* Synechococcus spongiarum” by BLASTx (>90% homology). Additional transcripts corresponding to carbon fixation via the Calvin-Benson-Bassham cycle were also present including glycoaldehydetransferase and ribulose-5-phosphate kinase.

*Host stress response*

Host transcripts involved in the cellular responses of the host to stress and/or bacteria or viruses that were homologous to *A. queenslandica* predicted genes were recovered. These included interleukin-1 receptor associated kinase 4-like (IRAK4) proteins (3 transcripts; 33-65% homology to *A. queenslandica* IRAK4), phosphatidylinositol-4,5-bisphosphate kinase 3-kinase (PIK; 10 transcripts, 63-92%), mitogen-activated port (1 transcript, 85%), NFκ-β (1 transcript, 93%), and Ras-related C3 botulinum toxin substrate 1-like (1 transcript, 90%). Other transcripts from the host relating to a variety of cellular activities, including apoptosis, were recovered (Caspase 8-like (4 transcripts, 36-50%), Sacsin-like (3 transcripts, 25-30%), AP-1 (2 transcripts, 52-68 %)).

**Reference**

Braker, G., Fesefeldt, A., and Witzel, K.-P. (1998). Development of PCR primer systems for amplification of nitrite reductase genes (*nir*K and *nir*S) to detect denitrifying bacteria in environmental samples. *Appl. Environ. Microbiol.* 64, 3769–3775.

Braker G, Tiedje JM (2003) Nitric Oxide Reductase (norB) Genes from Pure Cultures and Environmental Samples. *Appl. Environ. Microbiol.* 69, 3476–3483. doi: 10.1128/AEM.69.6.3476-3483.2003

Langmead B, Salzberg S. 2012. Fast gapped-read alignment with Bowtie 2. Nature Methods. 9: 357-359.


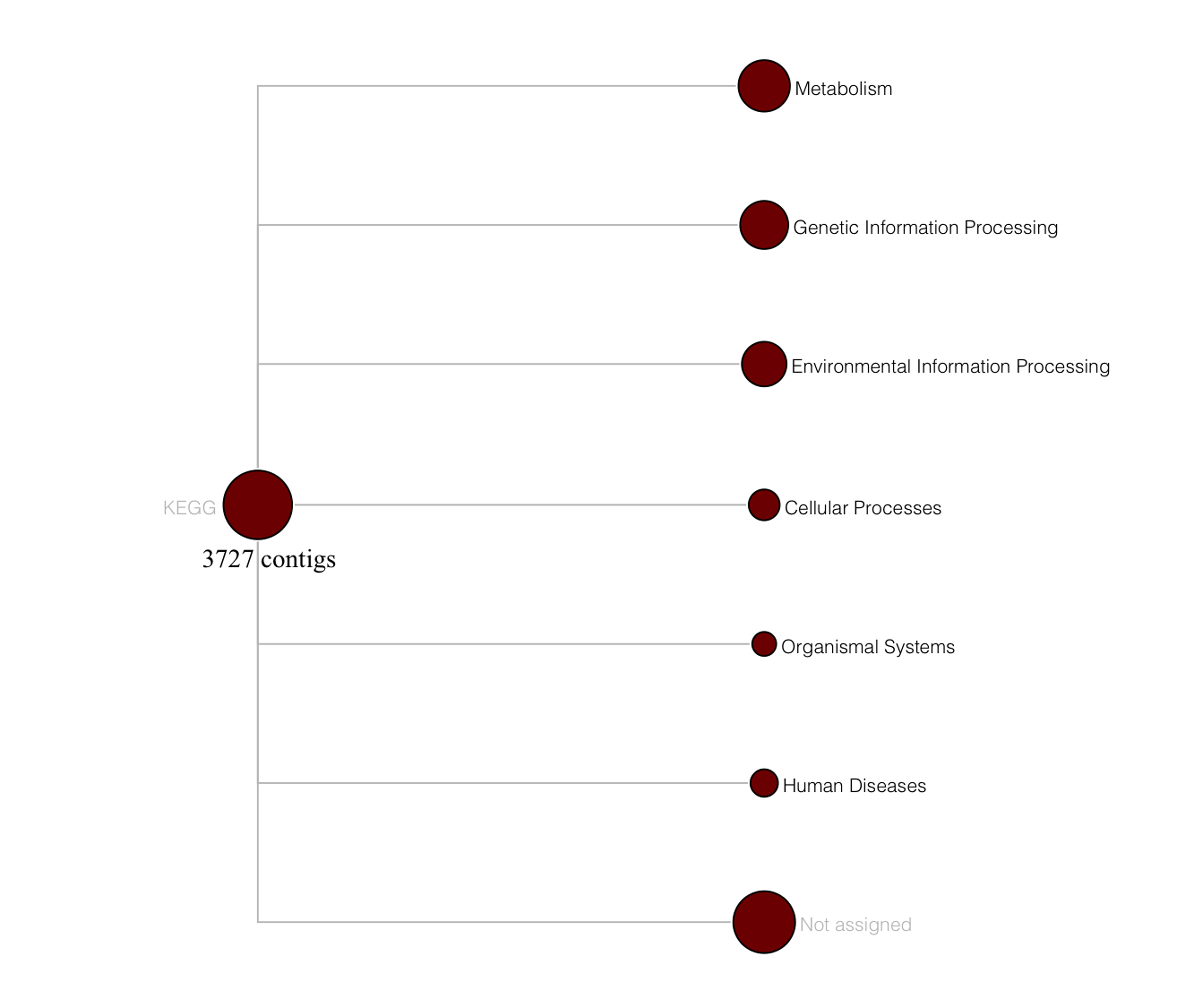


Figure S1. Distribution of prokaryotic transcriptional features in KEGG categories by the program MEGAN.


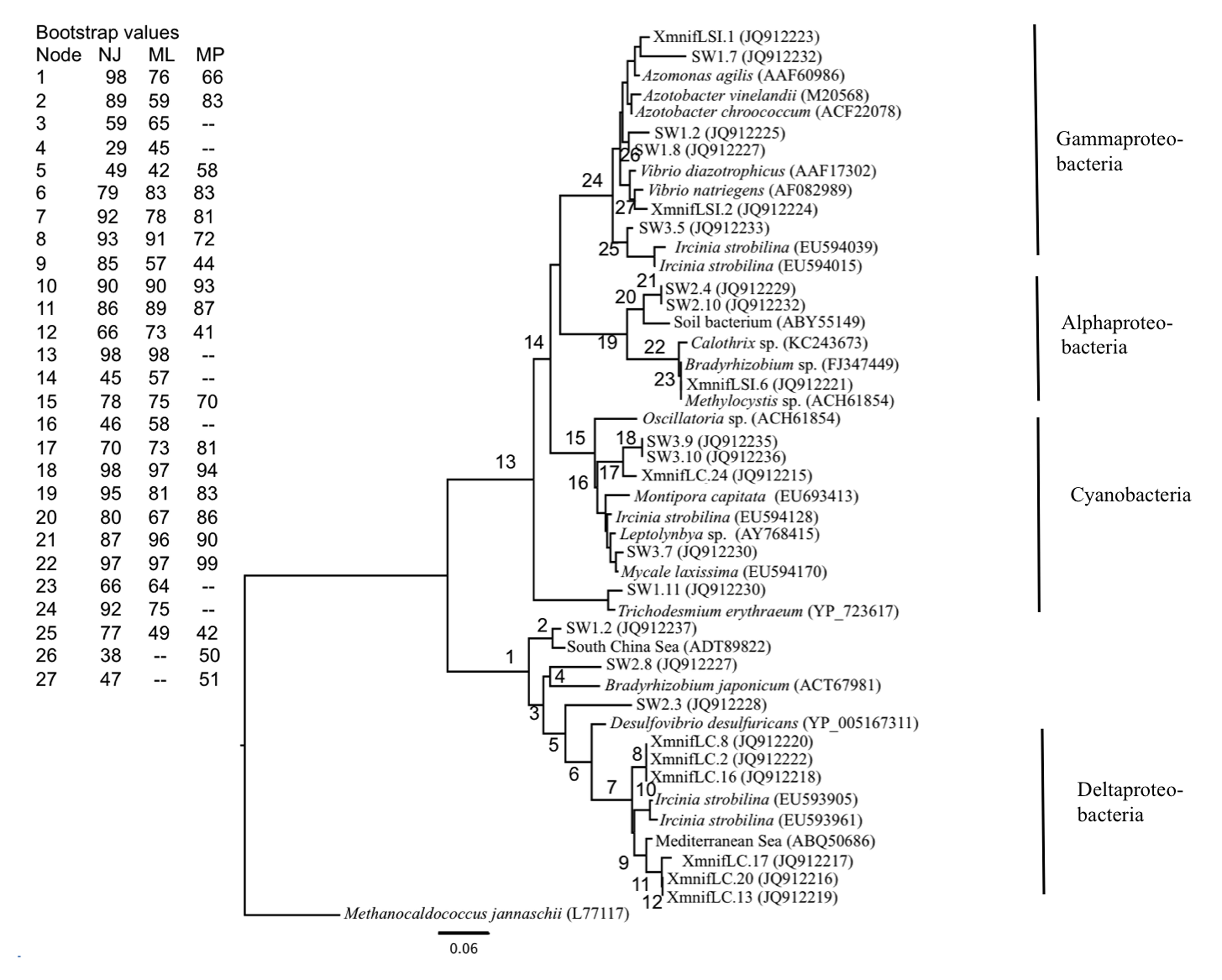


Figure S2. Neighbor-joining (NJ) tree based on *nif*H-deduced amino acid

sequences. *Methanocaldococcus jannaschii* was used as an outgroup to root the tree.

Sequences from this study are labeled as SW for seawater followed by clone number and

Xm for *Xestospongia muta* followed by the clone number. Bootstrap values are shown for nodes where at least one of the trees has a value above 50% for NJ, maxiumum parsimony (MP) or maximum likelihood (ML) versions of the tree.


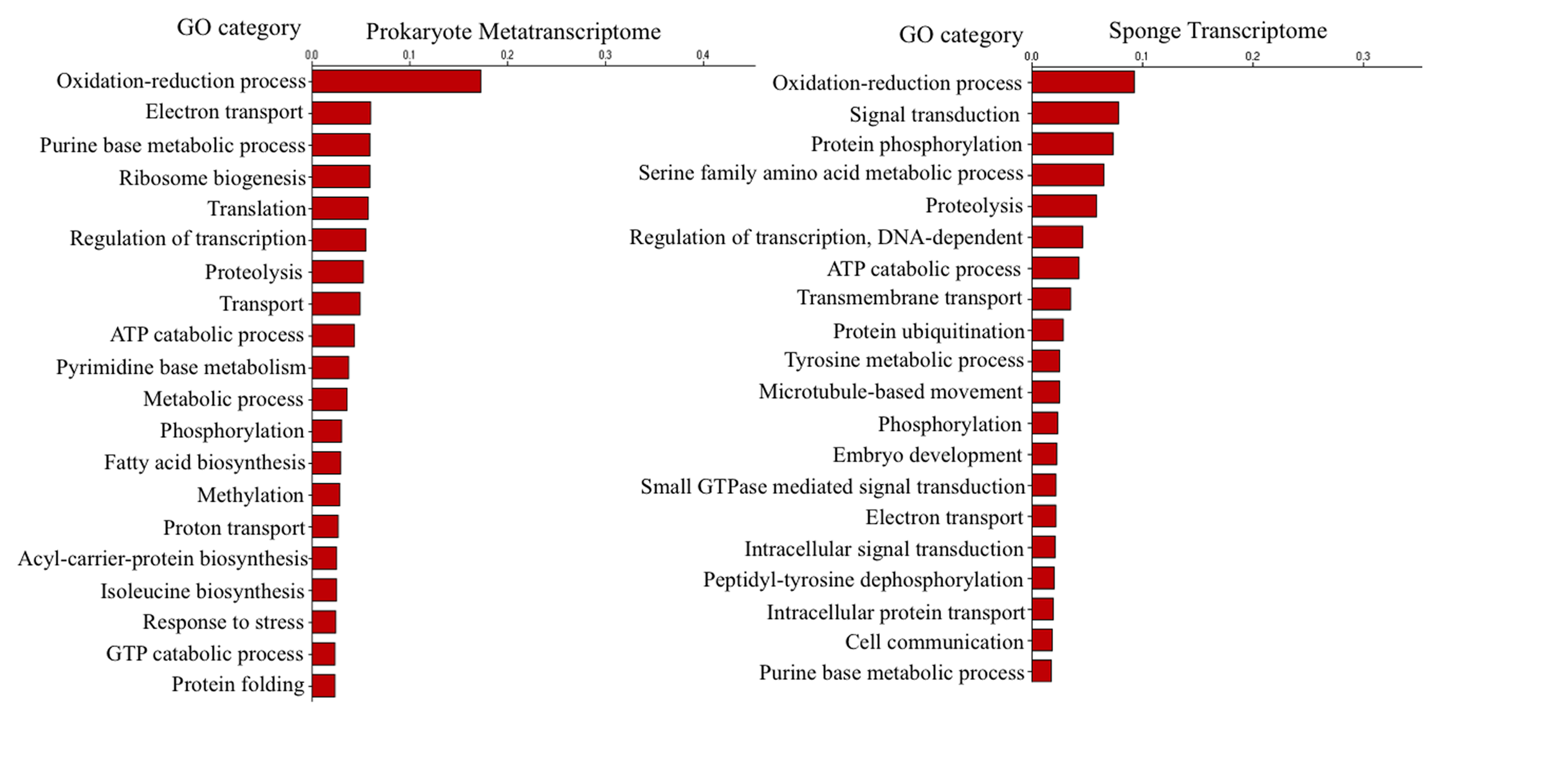


Figure S3. Gene ontology (GO) annotated transcirpts of the prokaryotic and sponge datasets. The 15 most abundant annotated transcripts are shown and FastAnnotator was used for annotations.


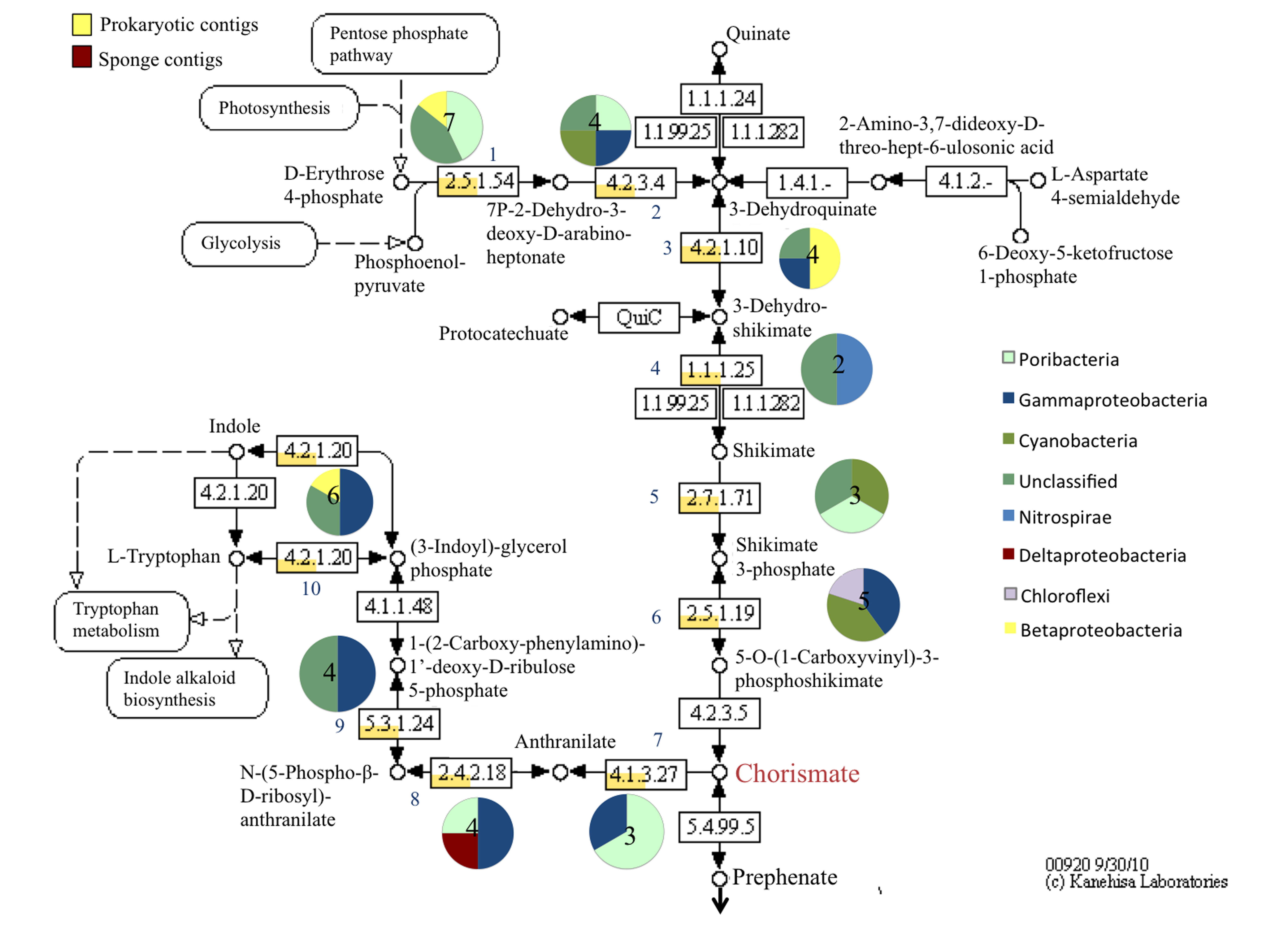


Figure S4. Relative abundance of prokaryotic and sponge transcripts involved in chorismate metabolism. MEGAN was used to visualize transcripts on the KEGG map. Pie charts near each enzyme number indicate the phyla represented by the transcripts and the number in the pie chart is the number of transcripts. A subset of the map is shown for clarity. Numbers correspond to the following enzymes: 1) 3-deoxy-7-phosphoheptulonate synthase 2) 3-dehydroquinate synthase 3) 3-dehydroquinate dehydratase 4) shikimate dehydrogenase 5) shikimate kinase 6) 3-phosphoshikimate 1-carboxyvinyltransferase 7) anthranilate synthase 8) amidophosphoribosyltransferase 9) N-acetylneuraminate epimerase 10) tryptophan synthase.


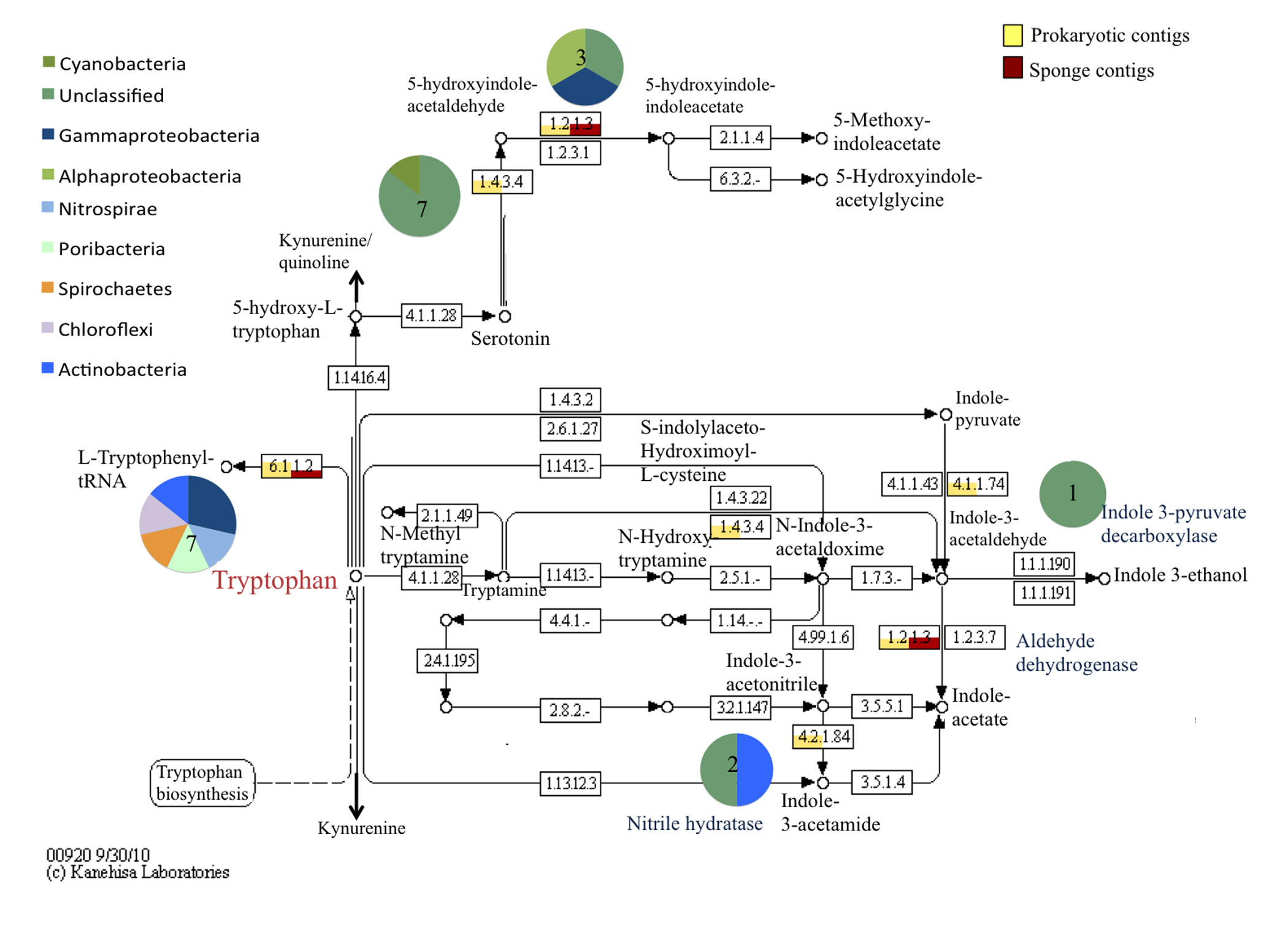


Figure S5. Relative abundance of prokaryotic and sponge transcripts involved in tryptophan metabolism. MEGAN was used to visualize transcripts on the KEGG map and only part of the map is shown for clarity. Pie charts near each enzyme number indicate the phyla represented by the transcripts and the number in the pie chart is the number of transcripts. Enzyme names are provided in blue. For clarity not all intermediates are shown.


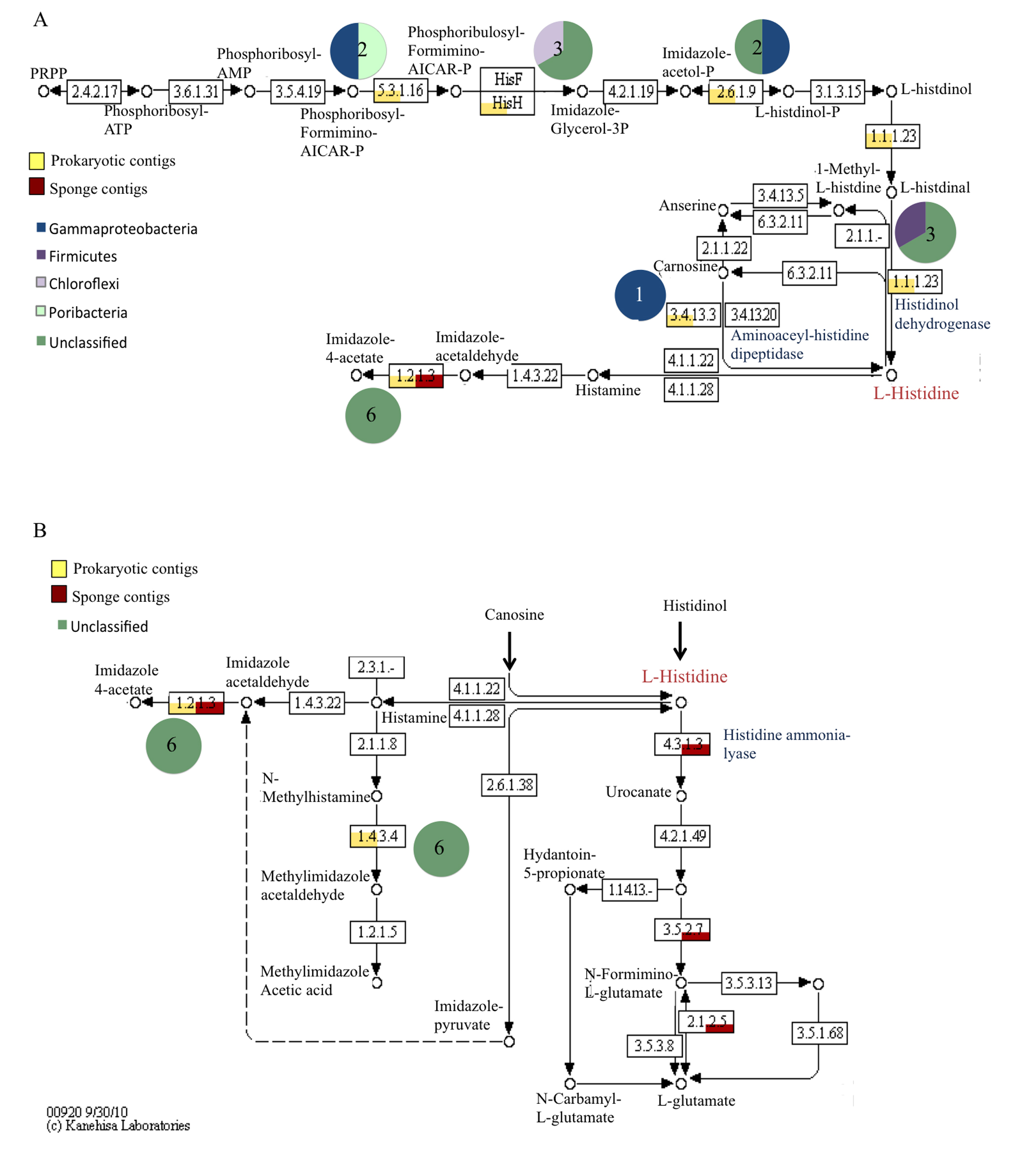


Figure S6. Relative abundance of prokaryotic and sponge transcripts involved in histidine metabolism. MEGAN was used to visualize transcripts on the KEGG map. The map was separated roughly into anabolic pathways (A) and catabolic pathways (B) for visualization. Pie charts near each enzyme number indicate the phyla represented by the transcripts and the number in the pie chart is the number of transcripts. Enzyme names are provided in blue. For clarity not all intermediates are shown.


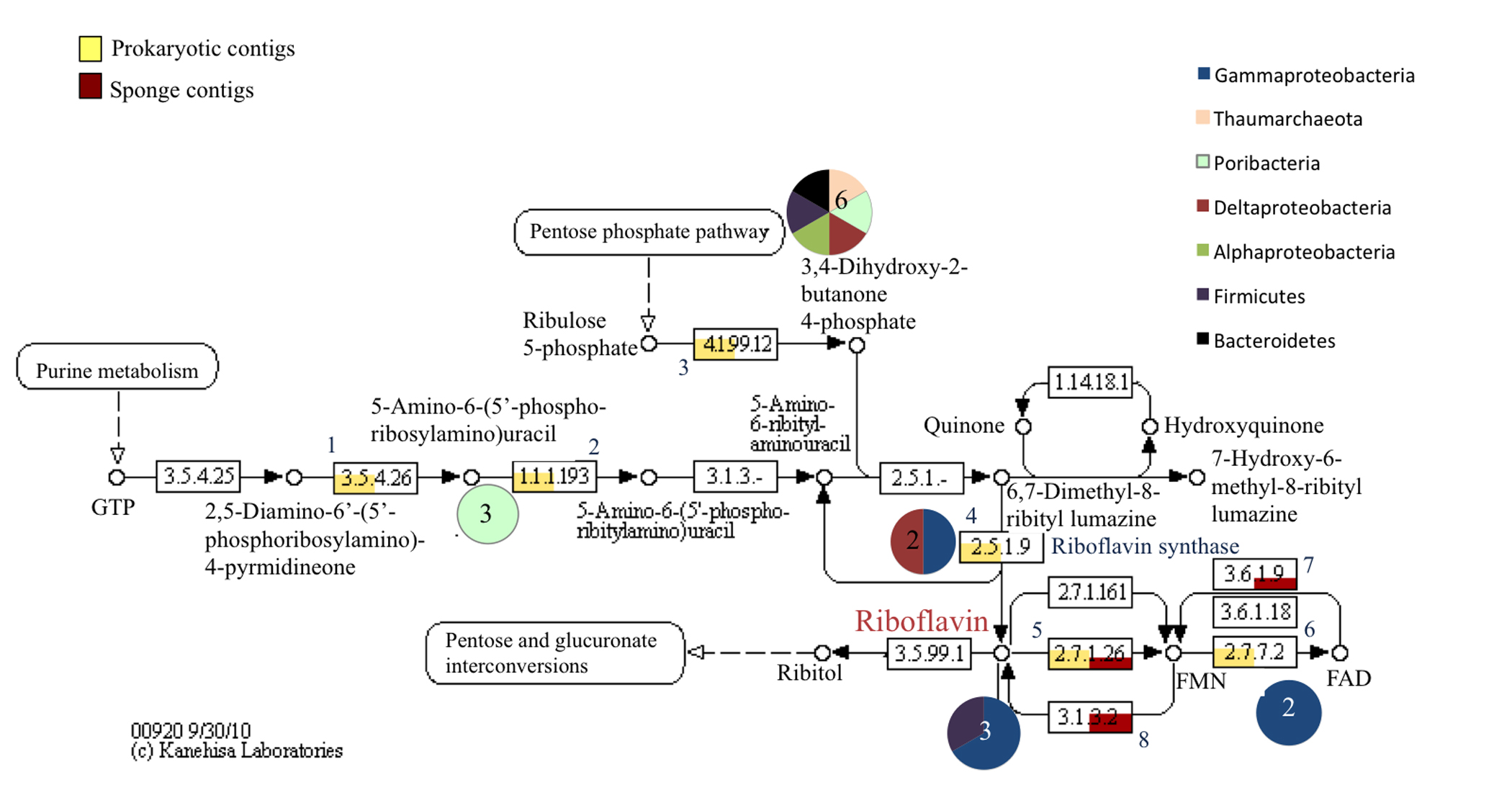


Figure S7. Relative abundance of prokaryotic and sponge transcripts involved in riboflavin metabolism. MEGAN was used to visualize transcripts on the KEGG map. Pie charts near each enzyme number indicate the phyla represented by the transcripts and the number in the pie chart is the number of transcripts. A subset of the map is shown for clarity. Numbers correspond to the following enzymes: 1) diaminohydroxyphosphoribosylaminopyrimidine deaminase 2) L-xylose 1-dehydrogenase 3) 3,4-dihydroxy-2-butanone-4-phosphate synthase 4) riboflavin synthase 5) riboflavin kinase 6) FAD synthetase 7) nucleotide diphosphatase 8) acid phosphatase.


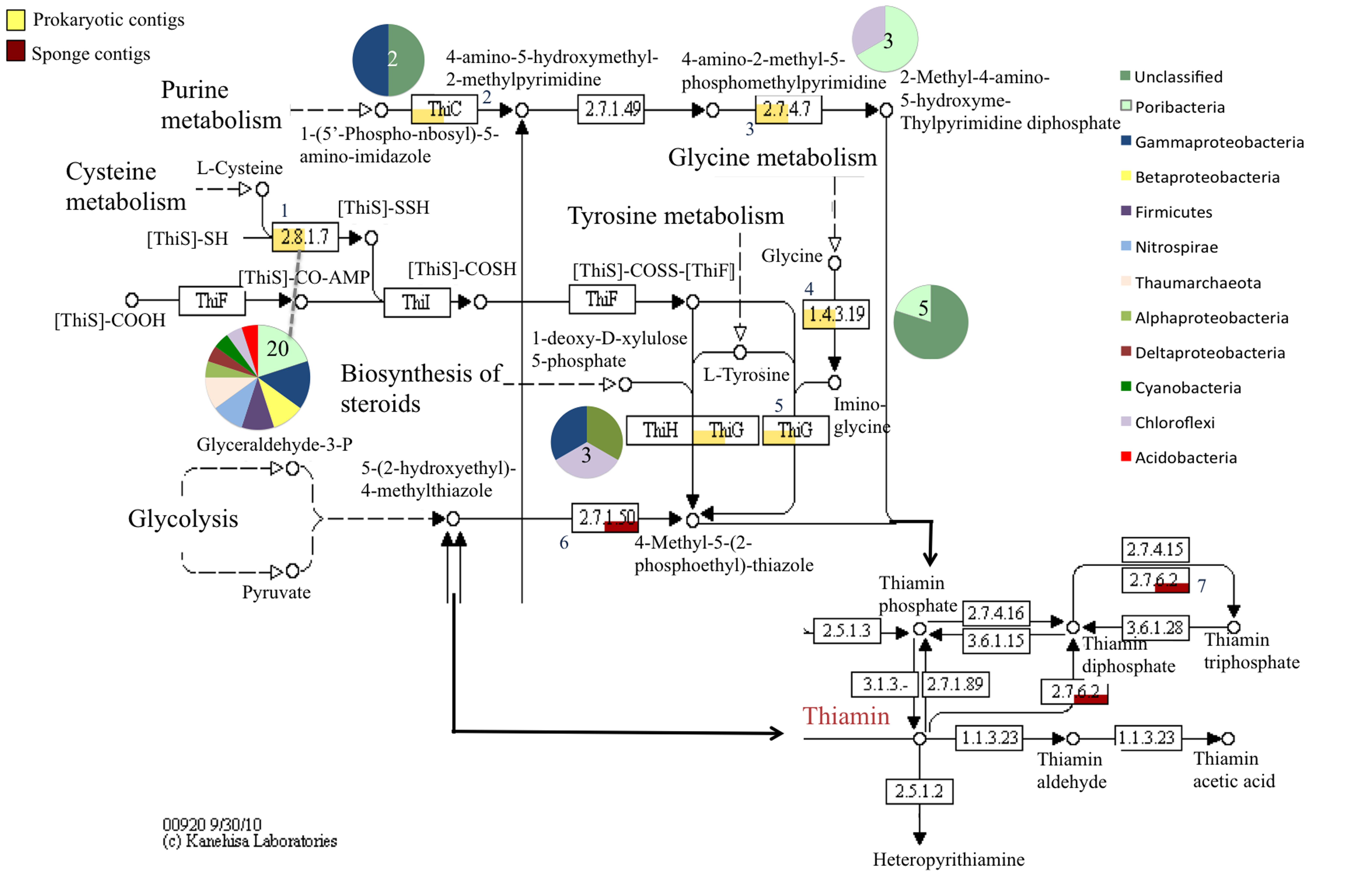


Figure S8. Relative abundance of prokaryotic and sponge transcripts involved in thiamin metabolism. MEGAN was used to visualize transcripts on the KEGG map. Pie charts near each enzyme number indicate the phyla represented by the transcripts and the number in the pie chart is the number of transcripts. A subset of the map is shown for clarity. Numbers correspond to the following enzymes: 1) cysteine desulfurase 2) thiamin biosynthesis protein 3) phosphomethylpyrimidine kinase 4) glycine oxidase 5) thiazole synthase 6) hydroxyethylthiazole kinase 7) thiamin diphosphokinase. For clarity not all intermediates are shown.

Table S1. Results of domain annotation of the prokaryotic transcripts using FastAnnotator. A subset of annotated transcripts are shown here, which are of interest in host-microbe interactions, but were not overlaid on a metabolic pathway map. Selected transcripts are those that were characterized with the eukaryotic-like domains of tetratricopeptide repeats (TPR), ankryin repeats (Ank), as well cobalamin biosynthesis transcripts (Cbi, Cob). A subset of TPR domains are shown; a total of 493 hits to TPR from 38 transcirpts were detected.

| **Query ID** | | **Domain Hit** | | **Domain short name** | | **Domain coverage** | | **E value** | |
| --- | --- | --- | --- | --- | --- | --- | --- | --- | --- |
| 13633_consensus | | pfam00515 | | TPR_1 | | 70.59% | | 2.00E-05 | |
| 15483_consensus | | pfam00515 | | TPR_1 | | 76.47% | | 0.006 | |
| 15998_consensus | | pfam00515 | | TPR_1 | | 76.47% | | 0.006 | |
| 18313_consensus | | pfam00515 | | TPR_1 | | 88.24% | | 3.00E-06 | |
| 18313_consensus | | pfam00515 | | TPR_1 | | 88.24% | | 3.00E-05 | |
| 18820_consensus | | pfam00515 | | TPR_1 | | 67.65% | | 0.008 | |
| 19122_consensus | | pfam00515 | | TPR_1 | | 97.06% | | 2.00E-06 | |
| 19122_consensus | | pfam00515 | | TPR_1 | | 91.18% | | 8.00E-06 | |
| 19122_consensus | | pfam00515 | | TPR_1 | | 97.06% | | 2.00E-05 | |
| 19122_consensus | | pfam00515 | | TPR_1 | | 88.24% | | 2.00E-05 | |
| 19122_consensus | | pfam00515 | | TPR_1 | | 91.18% | | 2.00E-05 | |
| 19122_consensus | | pfam00515 | | TPR_1 | | 97.06% | | 4.00E-05 | |
| 19122_consensus | | pfam00515 | | TPR_1 | | 94.12% | | 2.00E-04 | |
| 19122_consensus | | pfam00515 | | TPR_1 | | 94.12% | | 3.00E-04 | |
| 19122_consensus | | pfam00515 | | TPR_1 | | 85.29% | | 8.00E-04 | |
| 19381_consensus | | pfam00515 | | TPR_1 | | 94.12% | | 0.001 | |
| 21282_consensus | | pfam00515 | | TPR_1 | | 97.06% | | 3.00E-06 | |
| 22320_consensus | | pfam00515 | | TPR_1 | | 91.18% | | 1.00E-05 | |
| 22320_consensus | | pfam00515 | | TPR_1 | | 91.18% | | 1.00E-04 | |
| 26766_consensus | | pfam00023 | | Ank | | 81.82% | | 2.00E-04 | |
| 26766_consensus | | pfam00023 | | Ank | | 81.82% | | 9.00E-04 | |
| 26766_consensus | | pfam12796 | | Ank_2 | | 76.92% | | 5.00E-14 | |
| 26766_consensus | | pfam12796 | | Ank_2 | | 90.11% | | 1.00E-10 | |
| 26766_consensus | | pfam12796 | | Ank_2 | | 45.05% | | 2.00E-05 | |
| 26766_consensus | | pfam13606 | | Ank_3 | | 93.33% | | 0.004 | |
| 26766_consensus | | pfam13637 | | Ank_4 | | 96.30% | | 2.00E-05 | |
| 26766_consensus | | pfam13637 | | Ank_4 | | 68.52% | | 0.002 | |
| 26766_consensus | | pfam13857 | | Ank_5 | | 96.43% | | 4.00E-04 | |
| 26766_consensus | | pfam13857 | | Ank_5 | | 96.43% | | 0.002 | |
| 11358_consensus | | pfam01656 | | CbiA | | 59.91% | | 7.00E-12 | |
| 17524_consensus | | pfam01656 | | CbiA | | 84.33% | | 2.00E-17 | |
| 21628_consensus | | pfam01656 | | CbiA | | 53.00% | | 4.00E-06 | |
| 21629_consensus | | pfam01656 | | CbiA | | 52.07% | | 1.00E-04 | |
| 26871_consensus | | pfam01656 | | CbiA | | 80.18% | | 1.00E-17 | |
| 34500_consensus | | pfam01656 | | CbiA | | 50.69% | | 3.00E-10 | |
| 8117_consensus | | pfam01656 | | CbiA | | 87.56% | | 4.00E-20 | |
| **Query ID** | **Domain Hit** | | **Domain short name** | | **Domain coverage** | | **E value** | |  |
| 3633_consensus | | pfam02570 | | CbiC | | 89.90% | | 2.00E-64 | |
| 12768_consensus | | pfam01888 | | CbiD | | 29.12% | | 1.00E-09 | |
| 4561_consensus | | pfam01890 | | CbiG_C | | 93.33% | | 2.00E-20 | |
| 4561_consensus | | pfam11761 | | CbiG_mid | | 97.53% | | 3.00E-04 | |
| 14406_consensus | | pfam02571 | | CbiJ | | 13.01% | | 0.007 | |
| 4095_consensus | | pfam02571 | | CbiJ | | 21.95% | | 0.006 | |
| 15807_consensus | | pfam01891 | | CbiM | | 44.78% | | 6.00E-04 | |
| 20787_consensus | | pfam01891 | | CbiM | | 37.31% | | 0.001 | |
| 23732_consensus | | pfam01891 | | CbiM | | 56.72% | | 0.004 | |
| 8161_consensus | | pfam01891 | | CbiM | | 41.79% | | 0.003 | |
| 31302_consensus | | pfam01903 | | CbiX | | 88.68% | | 9.00E-23 | |
| 31302_consensus | | pfam01903 | | CbiX | | 37.74% | | 1.00E-07 | |
| 20280_consensus | | pfam02572 | | CobA_CobO_BtuR | | 36.05% | | 0.006 | |
| 34350_consensus | | pfam03186 | | CobD_Cbib | | 17.97% | | 0.002 | |
| 17900_consensus | | pfam02514 | | CobN-Mg_chel | | 8.43% | | 9.00E-37 | |
| 30920_consensus | | pfam02514 | | CobN-Mg_chel | | 20.76% | | 1.00E-80 | |
| 34384_consensus | | pfam02514 | | CobN-Mg_chel | | 12.79% | | 1.00E-36 | |
| 31748_consensus | | pfam02654 | | CobS | | 45.73% | | 0.007 | |
| 30460_consensus | | pfam02283 | | CobU | | 46.06% | | 0.009 | |

Table S2. Select annotations of poriferan-derived contigs using FastAnnotator. GO molecular function and domain annotations are given if they are available. Annotations reported here were selected because of their role in transport of vitamins and amino acids or host stress response.

| **Query ID** | **Best hit to nr** | **E-value** | **GO Molecular Function** | | **Domain** | |  |
| --- | --- | --- | --- | --- | --- | --- | --- |
| 12874_consensus | thiamine transporter 2-like | 2.27E-173 | GO:0005542 folic acid binding | GO:0008518 reduced folate carrier activity | | pfam01770 Folate_carrier | |  |
| 13674_consensus | riboflavin transporter 2-like | 1.18E-168 | GO:0032217 riboflavin transporter activity | | pfam06237 DUF1011 | |  |
| 13705_consensus | mitochondrial folate transporter/carrier-like | 4.59E-55 | GO:0008517 folic acid transporter activity | | pfam00153 Mito_carr | pfam09420 Nop16 | |  |
| 10619_consensus | proton-coupled amino acid transporter 1-like | 3.14E-109 | GO:0016799 hydrolase activity, hydrolyzing N-glycosyl compounds | GO:0003684 damaged DNA binding | GO:0008270 zinc ion binding | GO:0003906 DNA-(apurinic or apyrimidinic site) lyase activity | |  | |  |
| 10664_consensus | proton-coupled amino acid transporter 4-like | 2.85E-94 | GO:0046872 metal ion binding | GO:0015187 glycine transmembrane transporter activity | GO:0015193 L-proline transmembrane transporter activity | GO:0015180 L-alanine transmembrane transporter activity | GO:0015293 symporter activity | | pfam01490 Aa_trans | pfam12914 SH3_7 | |  |
| 31240_consensus | proton-coupled amino acid transporter 4-like | 8.03E-13 |  | |  | |  |
|  |  |  |  |  | |  | |

Table S2. Continued

| **Query ID** | **Best hit to nr** | **E-value** | **GO Molecular Function** | **Domain** |
| --- | --- | --- | --- | --- |
| 16011_consensus | proton-coupled amino acid transporter 4-like | 6.55E-174 |  | pfam01490 Aa_trans | pfam04116 FA_hydroxylase | pfam04093 MreD |
| 10619_consensus | proton-coupled amino acid transporter 1-like | 3.14E-109 | GO:0016799 hydrolase activity, hydrolyzing N-glycosyl compounds | GO:0003684 damaged DNA binding | GO:0008270 zinc ion binding | GO:0003906 DNA-(apurinic or apyrimidinic site) lyase activity |  |
| 31240_consensus | proton-coupled amino acid transporter 4-like | 8.03E-13 |  |  |
| 10619_consensus | proton-coupled amino acid transporter 1-like | 3.14E-109 | GO:0016799 hydrolase activity, hydrolyzing N-glycosyl compounds | GO:0003684 damaged DNA binding | GO:0008270 zinc ion binding | GO:0003906 DNA-(apurinic or apyrimidinic site) lyase activity |  |
| 16011_consensus | proton-coupled amino acid transporter 4-like | 6.55E-174 |  | pfam01490 Aa_trans | pfam04116 FA_hydroxylase | pfam04093 MreD |
| 16096_consensus | putative sodium-coupled neutral amino acid transporter 7-like | 4.03E-13 |  |  |
| 1646_consensus | putative sodium-coupled neutral amino acid transporter 10-like | 3.70E-96 |  | pfam01490 Aa_trans | pfam10277 Frag1 | pfam13042 |

Table S2. Continued

| **Query ID** | **Best hit to nr** | **E-value** | **GO Molecular Function** | **Domain** |
| --- | --- | --- | --- | --- |
| 16096_consensus | putative sodium-coupled neutral amino acid transporter 7-like | 4.03E-13 |  |  |
| 6086_consensus | putative sodium-coupled neutral amino acid transporter 7-like | 2.90E-144 |  | pfam01490 Aa_trans | pfam13520 AA_permease_2 |
| 9640_consensus | high affinity cationic amino acid transporter 1-like | 5.57E-225 | GO:0015171 amino acid transmembrane transporter activity | pfam13520 AA_permease_2 | pfam00324 AA_permease | pfam13906 AA_permease_C | pfam03595 C4dic_mal_tran | pfam01925 TauE | pfam13346 ABC2_membrane_5 | pfam13886 DUF4203 | pfam01061 ABC2_membrane | pfam01699 Na_Ca_ex | pfam06570 DUF1129 | pfam02674 Colicin_V |
| 20469_consensus | solute carrier family 2, facilitated glucose transporter member 1-like | 4.28E-109 | GO:0033300 dehydroascorbic acid transporter activity | GO:0003676 nucleic acid binding | GO:0055056 D-glucose transmembrane transporter activity |  |
| 20761_consensus | solute carrier organic anion transporter family member 4A1-like | 2.30E-166 | GO:0005215 transporter activity | pfam03137 OATP | pfam07690 MFS_1 | pfam13347 MFS_2 | pfam01943 Polysacc_synt | pfam04608 PgpA | pfam13886 |

Table S2. Continued

| **Query ID** | **Best hit to nr** | **E-value** | **GO Molecular Function** | **Domain** |
| --- | --- | --- | --- | --- |
| 16096_consensus | putative sodium-coupled neutral amino acid transporter 7-like | 4.03E-13 |  |  |
| 6086_consensus | putative sodium-coupled neutral amino acid transporter 7-like | 2.90E-144 |  | pfam01490 Aa_trans | pfam13520 AA_permease_2 |
| 9640_consensus | high affinity cationic amino acid transporter 1-like | 5.57E-225 | GO:0015171 amino acid transmembrane transporter activity | pfam13520 AA_permease_2 | pfam00324 AA_permease | pfam13906 AA_permease_C | pfam03595 C4dic_mal_tran | pfam01925 TauE | pfam13346 ABC2_membrane_5 | pfam13886 DUF4203 | pfam01061 ABC2_membrane | pfam01699 Na_Ca_ex | pfam06570 DUF1129 | pfam02674 Colicin_V |
| 20469_consensus | solute carrier family 2, facilitated glucose transporter member 1-like | 4.28E-109 | GO:0033300 dehydroascorbic acid transporter activity | GO:0003676 nucleic acid binding | GO:0055056 D-glucose transmembrane transporter activity |  |
| 20761_consensus | solute carrier organic anion transporter family member 4A1-like | 2.30E-166 | GO:0005215 transporter activity | pfam03137 OATP | pfam07690 MFS_1 | pfam13347 MFS_2 | pfam01943 Polysacc_synt | pfam04608 PgpA | pfam13886 DUF4203 |
|  |  |  |  |  |

Table S2. Continued

| **Query ID** | **Best hit to nr** | **E-value** | **GO Molecular Function** | **Domain** |
| --- | --- | --- | --- | --- |
| 30171_consensus | solute carrier organic anion transporter family member 4A1-like | 2.79E-27 | GO:0005215 transporter activity |  |
| 4613_consensus | solute carrier organic anion transporter family member 4A1-like | 1.56E-220 | GO:0015125 bile acid transmembrane transporter activity | pfam03137 OATP | pfam07690 MFS_1 | pfam07648 Kazal_2 | pfam00083 Sugar_tr |
| 6483_consensus | solute carrier organic anion transporter family member 4A1-like | 4.96E-160 | GO:0005215 transporter activity | pfam03137 OATP | pfam07690 MFS_1 |
| 5630_consensus | solute carrier organic anion transporter family member 4A1-like | 2.23E-104 | GO:0005215 transporter activity |  |
| 33213_consensus | canalicular multispecific organic anion transporter 2-like, partial | 1.18E-92 | GO:0042626 ATPase activity, coupled to transmembrane movement of substances | GO:0005524 ATP binding | pfam00664 ABC_membrane |
| 6034_consensus | solute carrier family 2, facilitated glucose transporter member 1-like | 2.12E-106 | GO:0033300 dehydroascorbic acid transporter activity | GO:0055056 D-glucose transmembrane transporter activity | GO:0005351 sugar:hydrogen symporter activity | pfam01943 Polysacc_synt | pfam00902 TatC | pfam12730 ABC2_membrane_4 |
| 6646_consensus | putative glycerol-3-phosphate transporter 3-like | 3.04E-85 | GO:0005351 sugar:hydrogen symporter | pfam07690 MFS_1 |

Table S2. Continued

| **Query ID** | **Best hit to nr** | **E-value** | **GO Molecular Function** | **Domain** |
| --- | --- | --- | --- | --- |
| 6646_consensus | putative glycerol-3-phosphate transporter 3-like | 3.04E-85 | GO:0005351 sugar:hydrogen symporter activity | pfam07690 MFS_1 |
| 4639_consensus | acetyl-coenzyme A transporter 1-like | 2.54E-181 | GO:0008521 acetyl-CoA transporter activity | pfam13000 Acatn |
| 2683_consensus | choline transporter-like protein 2-like | 0 |  | pfam04515 Choline_transpo | pfam04610 TrbL |
| 2479_consensus | UDP-N-acetylglucosamine transporter-like | 8.45E-48 | GO:0046872 metal ion binding | GO:0003676 nucleic acid binding | GO:0005338 nucleotide-sugar transmembrane transporter activity | GO:0005351 sugar:hydrogen symporter activity | pfam04588 HIG_1_N |
| 12021_consensus | UDP-N-acetylglucosamine/UDP-glucose/GDP-mannose transporter-like | 8.64E-93 |  | pfam03151 TPT |
| 10678_consensus | proton myo-inositol cotransporter-like | 3.58E-224 | GO:0022891 substrate-specific transmembrane transporter activity | GO:0032440 2-alkenal reductase [NAD(P)] activity | pfam00083 Sugar_tr | pfam07690 MFS_1 | pfam13347 MFS_2 |
| 13696_consensus | ammonium transporter Rh type B-like | 1.28E-07 | GO:0008519 ammonium transmembrane transporter activity |  |

Table S2. Continued

| **Query ID** | **Best hit to nr** | **E-value** | **GO Molecular Function** | **Domain** |
| --- | --- | --- | --- | --- |
| 16023_consensus | ammonium transporter 1 member 1-like | 5.99E-27 | GO:0046872 metal ion binding | GO:0003874 6-pyruvoyltetrahydropterin synthase activity | GO:0008519 ammonium transmembrane transporter activity |  |
| 4338_consensus | ammonium transporter Rh type B-like | 9.23E-179 | GO:0008519 ammonium transmembrane transporter activity | GO:0030506 ankyrin binding | GO:0051739 ammonia transmembrane transporter activity |  |
| 620_consensus | ammonium transporter 1 member 1-like | 2.34E-40 | GO:0003676 nucleic acid binding | GO:0046872 metal ion binding | GO:0008519 ammonium transmembrane transporter activity | GO:0005524 ATP binding | GO:0000155 two-component sensor activity |  |
| 9507_consensus | putative transporter arsB-like | 6.88E-115 | GO:0015105 arsenite transmembrane transporter activity |  |
| 9533_consensus | putative transporter arsB-like | 2.94E-99 | GO:0015105 arsenite transmembrane transporter activity |  |
| 6617_consensus | putative transporter arsB-like | 1.89E-117 | GO:0015105 arsenite transmembrane transporter activity |  |
| 12939_consensus | heat shock 70 kDa protein 12A-like | 6.04E-76 |  |  |

Table S2. Continued

| **Query ID** | **Best hit to nr** | **E-value** | **GO Molecular Function** | **Domain** |
| --- | --- | --- | --- | --- |
| 10320_consensus | activator of 90 kDa heat shock protein ATPase homolog 1 | 4.00E-08 | GO:0051087 chaperone binding | GO:0001671 ATPase activator activity |  |
| 10548_consensus | heat shock 70 kDa protein 12A-like, partial | 1.63E-123 |  |  |
| 1294_consensus | heat shock 70 kDa protein 12A-like | 2.09E-170 | GO:0005524 ATP binding |  |
| 13903_consensus | heat shock 70 kDa protein 12A-like | 1.36E-46 |  |  |
| 15469_consensus | heat shock 70 kDa protein 12A-like, partial | 1.18E-48 |  |  |
| 15471_consensus | heat shock 70 kDa protein 12A-like, partial | 8.29E-62 |  |  |
| 15789_consensus | heat shock 70 kDa protein 12A-like | 1.22E-36 |  |  |
| 1595_consensus | heat shock 70 kDa protein 12A-like, partial | 2.26E-97 |  |  |
| 18326_consensus | heat shock 70 kDa protein 14-like | 1.09E-89 | GO:0032440 2-alkenal reductase [NAD(P)] activity | GO:0005524 ATP binding |  |
| 19790_consensus | heat shock 70 kDa protein 12B-like | 1.01E-44 |  | pfam05565 Sipho_Gp157 |
| 21152_consensus | heat shock 70 kDa protein 12A-like, partial | 7.01E-24 |  |  |
| 21352_consensus | heat shock 70 kDa protein 12A-like, partial | 1.94E-58 |  |  |

Table S2. Continued

| **Query ID** | **Best hit to nr** | **E-value** | **GO Molecular Function** | **Domain** |
| --- | --- | --- | --- | --- |
| 2463_consensus | heat shock protein 105 kDa-like | 7.59E-130 | GO:0005524 ATP binding | GO:0032440 2-alkenal reductase [NAD(P)] activity |  |
| 2496_consensus | 10 kDa heat shock protein, mitochondrial-like | 6.47E-44 | GO:0005524 ATP binding | pfam00166 Cpn10 |
| 2779_consensus | heat shock 70 kDa protein 12A-like | 8.42E-70 |  |  |
| 3133_consensus | heat shock 70 kDa protein 12A-like, partial | 1.11E-39 |  |  |
| 319_consensus | heat shock 70 kDa protein 12A-like | 1.02E-117 |  |  |
| 3266_consensus | heat shock protein 105 kDa-like | 1.42E-28 | GO:0005524 ATP binding |  |
| 344_consensus | heat shock 70 kDa protein 12A-like, partial | 4.35E-102 |  |  |
| 3469_consensus | 60 kDa heat shock protein, mitochondrial-like | 6.05E-266 | GO:0002039 p53 binding | GO:0015299 solute:hydrogen antiporter activity | GO:0051087 chaperone binding | GO:0005524 ATP binding | pfam00118 Cpn60_TCP1 |
| 3981_consensus | heat shock 70 kDa protein 12A | 5.57E-263 |  |  |
| 4634_consensus | heat shock 70 kDa protein 12A-like | 3.35E-100 | GO:0005524 ATP binding |  |
| 5875_consensus | heat shock 70 kDa protein 12A-like, partial | 3.91E-78 |  |  |

Table S2. Continued

| **Query ID** | **Best hit to nr** | **E-value** | **GO Molecular Function** | **Domain** |
| --- | --- | --- | --- | --- |
| 6429_consensus | heat shock 70 kDa protein 12A-like | 4.37E-71 |  |  |
| 7277_consensus | heat shock cognate 70 kDa protein 1-like | 3.71E-286 | GO:0000175 3'-5'-exoribonuclease activity | GO:0003723 RNA binding | GO:0019899 enzyme binding | pfam00012 HSP70 | pfam10712 NAD-GH | pfam06723 MreB_Mbl |
| 8201_consensus | heat shock 70 kDa protein 12A | 4.08E-130 |  |  |
| 9323_consensus | heat shock 70 kDa protein 12A-like | 6.54E-70 | GO:0005524 ATP binding |  |
| 9859_consensus | heat shock 70 kDa protein 12A-like | 4.28E-38 |  |  |
| 10320_consensus | activator of 90 kDa heat shock protein ATPase homolog 1 | 4.00E-08 | GO:0051087 chaperone binding | GO:0001671 ATPase activator activity |  |
